# Plant Ribosomal Proteins, RPL12 and RPL19, Play a Role in Nonhost Disease Resistance against Bacterial Pathogens

**DOI:** 10.3389/fpls.2015.01192

**Published:** 2016-01-06

**Authors:** Satish Nagaraj, Muthappa Senthil-Kumar, Vemanna S. Ramu, Keri Wang, Kirankumar S. Mysore

**Affiliations:** ^1^Plant Biology Division, The Samuel Roberts Noble Foundation Inc.Ardmore, OK, USA; ^2^National Institute of Plant Genome ResearchNew Delhi, India

**Keywords:** nonhost resistance, VIGS, ribosomal proteins, *Nicotiana benthamiana*, hypersensitive response, plant defense

## Abstract

Characterizing the molecular mechanism involved in nonhost disease resistance is important to understand the adaptations of plant-pathogen interactions. In this study, virus-induced gene silencing (VIGS)-based forward genetics screen was utilized to identify genes involved in nonhost resistance in *Nicotiana benthamiana*. Genes encoding ribosomal proteins, RPL12 and RPL19, were identified in the screening. These genes when silenced in *N. benthamiana* caused a delay in nonhost bacteria induced hypersensitive response (HR) with concurrent increase in nonhost bacterial multiplication. Arabidopsis mutants of *AtRPL12* and *AtRPL19* also compromised nonhost resistance. The studies on *NbRPL12* and *NbRPL19* double silenced plants suggested that both RPL12 and RPL19 act in the same pathway to confer nonhost resistance. Our work suggests a role for RPL12 and RPL19 in nonhost disease resistance in *N. benthamiana* and Arabidopsis. In addition, we show that these genes also play a minor role in basal resistance against virulent pathogens.

## Introduction

Disease resistance mechanisms of plants are continuously evolving for the sole purpose of negating the attempted infections of the ever adapting pathogens. The well-studied resistance (*R*)-gene mediated disease resistance is often very specific to a particular plant genotype or cultivar and a particular race of a pathogen. In contrast, nonhost resistance can act against all races of a particular pathogen and can occur in all cultivars of a host plant species. For many years, several aspects of plant disease resistance mechanisms and the adaptation of pathogens to overcome the plant defense have been studied and the resistance pathways have been elucidated (Abramovitch and Martin, 2004; Block et al., 2008). In addition to this, plants also respond to pathogen infection by a weak and generic response called as basal resistance (Senthil-Kumar and Mysore, 2013). This defense response reduces the virulent pathogen growth and may also delay the disease development.

Nonhost resistance mechanisms are not yet fully understood (Heath, 2000; Thordal-Christensen, 2003; Mysore and Ryu, 2004; Senthil-Kumar and Mysore, 2013). In some instances, it is suggested that plant responses toward host and nonhost pathogens trigger similar defense responses that include preformed defenses, inducible defenses, and signaling (Thordal-Christensen, 2003; Mysore and Ryu, 2004; Gill et al., 2015). However, the end result of host and nonhost resistance are different where in the host resistance confers resistance only against pathogen isolates that have the corresponding avirulence gene while the nonhost resistance confers resistance against all isolates of a particular pathogen (Heath, 2000; Thordal-Christensen, 2003; Mysore and Ryu, 2004; Niks and Marcel, 2009; Senthil-Kumar and Mysore, 2013; Gill et al., 2015). Understanding nonhost resistance mechanisms is therefore important to engineer plants for durable resistance.

Previous studies have mainly used *Pseudomonas*-Arabidopsis interactions to characterize nonhost resistance against bacterial pathogens. We and others have recently used virus-induced gene silencing (VIGS) as a tool in *Nicotiana benthamiana* to identify plant genes involved in nonhost resistance (Peart et al., 2002; Kanzaki et al., 2003; Rojas et al., 2012; Senthil-Kumar and Mysore, 2012; Wang et al., 2012; Senthil-Kumar et al., 2013). In addition, other groups have also used VIGS to understand the role of plant genes in defense responses. For example, 192 Avr4-responsive tomato cDNA fragments (*ART*) were analyzed for their role in hypersensitive response (HR) by utilizing VIGS in *N. benthamiana* and this study attributed a role for ribosomal protein 19 (RPL19) (Gabriëls et al., 2006).

RPLs are the components of the ribosome machinery and, to a certain extent, are required for protein synthesis. A component of large subunit of ribosome L19, though not important for translation, is important to interlink the large and small subunits. L19 along with L14 interacts with L3 and rRNA elements of large and small subunits and is suspected to increase the stability of the inter subunit bridges (Harms et al., 2001). L19 along with a calmodulin-like protein is required for regulation of protein synthesis during photosynthetic carbon assimilation in tobacco (Mönke and Sonnewald, 1995). L12 proteins, often found as a L7/L12 dimer, are the only multicopy ribosomal proteins and are involved in the regulation of protein synthesis (Grebenyuk et al., 2009). L7 and L12 are transcribed from the same gene and they differ by aminoacetylation of a serine residue at the N-terminal end of the L7 protein (Bailey-Serres et al., 1997).

Though ribosomal proteins are components of translational machinery, these proteins are suspected to have extra ribosomal functions such as stress signaling (Wool, 1996; Warner and McIntosh, 2009). Upon screening the large subunit proteins, it was shown that RPL19 had the highest RNA chaperone activity when tested splicing of the *Thymidylate Synthase* gene (Semrad et al., 2004). In addition, the protein chaperone activity of RPL19 protein was confirmed by measuring its activity with substrates that included alcohol dehydrogenase and lysozyme (Kovacs et al., 2009). Similar to RPL19 RNA splicing activity, RPL12 is shown to be involved in autoregulation of mRNA splicing in *Caenorhabditis elegans* (Mitrovich and Anderson, 2000).

Extraribosomal functions of ribosomal proteins related to disease resistance or stress have been reported in recent years. A truncated RPL3 protein from yeast was transformed into tobacco and was shown to be involved in disease resistance (Di and Tumer, 2005). The endogenous RPL3 protein levels were high in transgenic plants expressing high levels of full length or truncated RPL3. This conferred resistance against mycotoxin deoxynivalenol (Di and Tumer, 2005). By selectively mutating plastid ribosomal proteins in tobacco, it was shown that RPL33 was non-essential for normal growth but was required for resistance to chilling stress (Rogalski et al., 2008).

In order to identify and characterize the genes involved in nonhost resistance, our laboratory utilized the VIGS approach to screen a normalized *N. benthamiana* cDNA library and identified *NbRPL12* and *NbRPL19* genes that encode ribosomal proteins L12 and L19, respectively. These genes when silenced in *N. benthamiana* compromised nonhost resistance and HR induced by nonhost pathogens. Arabidopsis mutants for *AtRPL12* and *AtRPL19* also compromised nonhost resistance.

## Results

### VIGS-based nonhost screen identifies *NbRPL12* and *NbRPL19*

To identify plant genes involved in nonhost disease resistance, clones from a normalized *N. benthamiana* cDNA library (Anand et al., 2007; Senthil-Kumar et al., 2013) were individually silenced using *Tobacco rattle virus* (TRV)-based VIGS (Senthil-Kumar and Mysore, 2014) and screened for altered HR to nonhost pathogen infection. Upon inoculation with a nonhost pathogen, *P. syringae* pv. tomato T1, the control plants (TRV2::*GFP*; *GFP* sequence does not have any homology to plant DNA and therefore will not cause gene silencing) showed a typical HR characterized by necrosis limited around the inoculation site as early as 1 day post inoculation (dpi), while in some silenced plants delay in HR or development of disease symptoms were observed. In this manuscript we focus on two of such clones, TRV2::*14G03* and TRV2::*19A05* that showed delay in nonhost HR upon silencing in *N. benthamiana*. VIGS caused 40 and 80 percent down-regulation of target gene expression in TRV2::*14G03* and TRV2::*19A05* inoculated plants, respectively, as demonstrated by quantitative RT-PCR (RT-qPCR; Supplementary Figure S1). Inserts in *14G03* and *19A05* cDNA clones were sequenced and the resulting sequences were used for BLAST search against the NCBI database and determined the identity of gene sequences as *RPL12* (NCBI accession # JZ764382) and *RPL19* (NCBI accession # JZ764609), respectively. A BLAST search using the currently available *N. benthamiana* genomic sequence (http://www.sc.noble.org/niben/blast.php) indicated that *NbRPL12* and *NbRPL19* has 3 and 9 copies, respectively.

### *NbRPL12* and *NbRPL19* silenced plants show a delay in nonhost pathogen induced HR

**Figure 1:**
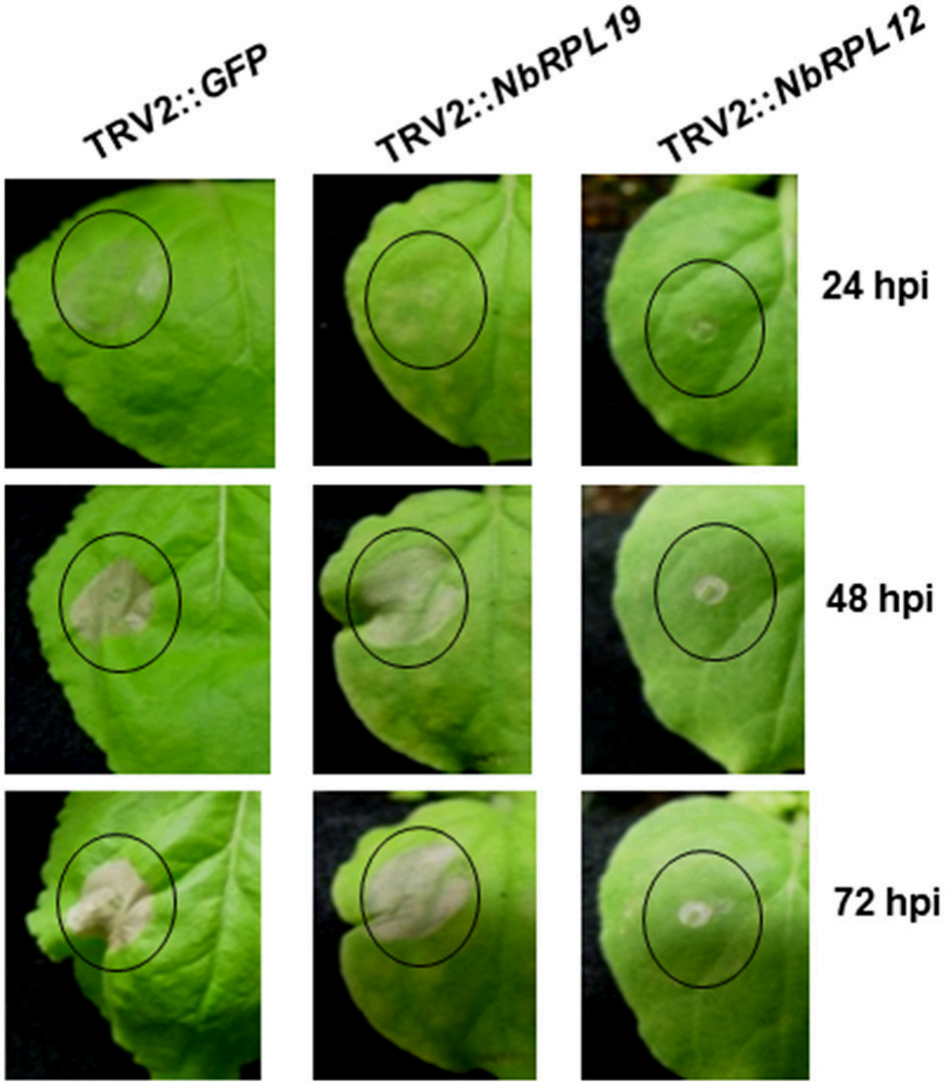
**Silencing of ***NbRPL12*** and ***NbRPL19*** genes in ***N. benthamiana*** delayed development of HR in nonhost pathogen inoculated leaves**. Using TRV-based VIGS, *N. benthamiana* plants were silenced for *NbRPL12* or *NbRPL19*. Abaxial side of silenced and control (TRV2::*GFP*) leaves were inoculated (OD_600_ = 0.001) using needleless syringe with nonhost pathogen *P. syringae* pv. tomato T1 (solid circles). The delay in HR symptoms were photographed at different days post inoculation. Similar response was observed in three individual experiments that included five biological replicates.

We verified the delayed HR phenotype in *NbRPL12*- and *NbRPL19*-silenced plants by inoculating a nonhost pathogen in a separate experiment. Upon infiltration of nonhost pathogen, *P. syringae* pv. tomato T1, we observed nonhost HR in non-silenced control plants (Figure 1). However, both *NbRPL12* and *NbRPL19*-silenced plants showed delay in HR at 24 hpi (Figure 1). The delay in HR was more drastic in *NbRPL12* silenced plants than *NbRPL19* silenced plants (Figure 1).

To analyze if the delay in HR also occurs during gene-for-gene mediated resistance and due to chemicals, *N. benthamiana* plants silenced for *RPL12* or *RPL19* were either inoculated with pathogen elicitors/effectors and corresponding plant receptors to induce HR due to gene-for-gene interactions (Supplementary Figure S2) or treated with various cell death causing chemicals (Supplementary Figure S3). We used a mixture of *Agrobacterium* carrying either *Pro35S:tvEIX* & *Pro35S:LeEix2* or *Pro35S:AvrPto* & *Pro35S:Pto* or *Pro35S:Avr9* & *Pro35S:Cf9* constructs for testing gene-for-gene induced HR. Ethylene inducing xylanase (EIX) and Avirulent 9 (Avr9) are fungal effectors. AvrPto is a bacterial effector. LeEix2, Cf9 and Pto are cognate R-proteins that can recognize these effectors, respectively. In addition, general cell death inducing chemicals such as ethanol, NaCl and H_2_O_2_ were used to assess the cell death response in *NbRPL12* and *NbRPL19* silenced plants. In all cases tested, the silenced plants caused HR similar to that of wild-type or vector control. Overall these results suggest that the delay in cell death is not a general response of the silenced plant (Supplementary Figures S2, S3).

### *NbRPL12* and *NbRPL19* silenced plants have increased nonhost pathogen mutliplication

**Figure 2:**
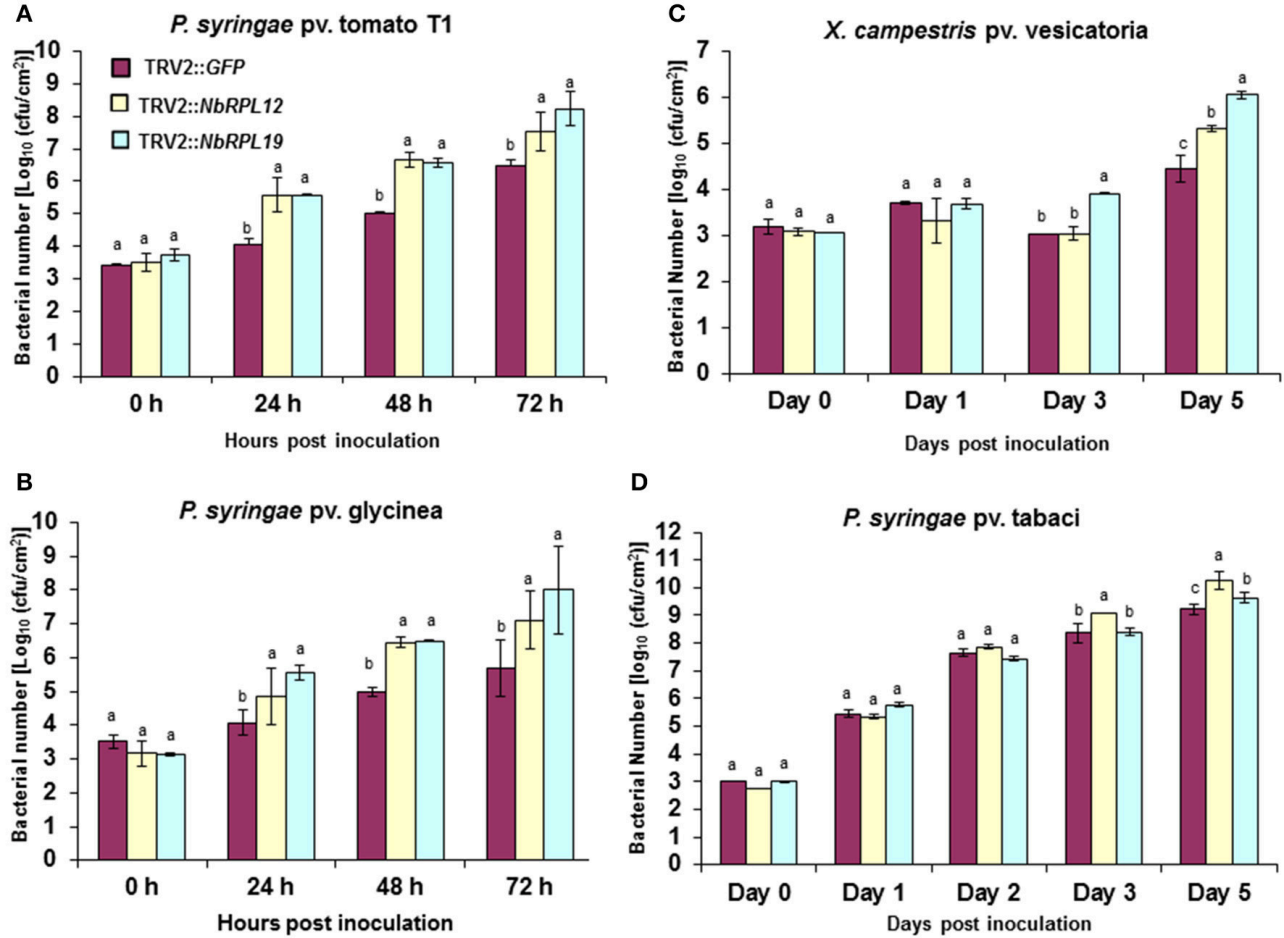
**Multiplication of host and nonhost pathogens in ***NbRPL12*** and ***NbRPL19*** gene silenced ***N. benthamiana*** leaves**. VIGS was performed using TRV2::*NbRPL12* and TRV2::*NbRPL19* constructs on 3 week old *N. benthamiana* plants. Plants inoculated with TRV2::*GFP* were used as vector control. The silenced leaves were inoculated using needleless syringe with nonhost pathogens, *P. syringae* pv. tomato T1 (**A**, OD_600_ = 0.0002), *P. syringae* pv. glycinea (**B**, OD_600_ = 0.001) and *X. campestris* pv. vesicatoria (**C**, OD_600_ = 0.0002) or host pathogen, *P. syringae* pv. tabaci (**D**, OD_600_ = 0.0001). Bacterial multiplication of host or nonhost pathogens was quantified at various time intervals. The error bars represent standard deviation for three replications. Different letters above the bars indicate a significant difference from Two-way ANOVA at *p* < 0.05 with Tukey's honest significant differences (HSD) means separation test (α = 0.05) among control and different gene silenced plants within a time point. See Supplementary Table S2 for details about statistics.

*TRV2::NbRPL12* and *TRV2::NbRPL19* inoculated *N. benthamiana* plants along with vector control (TRV2::*GFP* inoculated) plants, were individually infiltrated using a needless syringe with either host (*P. syringae* pv. tabaci) or nonhost bacterial pathogens (*P. syringae* pv. tomato T1, *P. syringae* pv. glycinea and *Xanthomonas campestris* pv. vesicatoria) (Figure 2). There was a significant increase in nonhost bacterial multiplication in the *NbRPL12* and *NbRPL19* silenced plants when compared to non-silenced vector control plants (Figures 2A–C). There was more than 10-fold increase in *P. syringae* pv. tomato T1 population in the silenced plants than that of vector control plants in all time points tested (Figure 2A). Similarly, the population of other nonhost pathogens, *P. syringae* pv. glycinea or *X. campestris* pv. vesicatoria, in the *NbRPL19* silenced plants, was increased by more than 10-fold (Figures 2B,C). The bacterial titer of *P. syringae* pv. glycinea and *X. campestris* pv. vesicatoria in *NbRPL12* silenced plants was increased to a lesser extent compared to *NbRPL19* silenced plants. Taken together, these bacterial growth assays suggest that *NbRPL12* and *NbRPL19* silenced plants are compromised for nonhost disease resistance, thereby allowing the multiplication and accumulation of nonhost bacteria.

When the host pathogen *P. syringae* pv. tabaci was infiltrated into leaves of *NbRPL12* and *NbRPL19* silenced plants they were able to accumulate more than three- and two-fold higher, respectively, when compared to vector control plants at 5 dpi (Figure 2D). These results suggest that *NbRPL12* and *NbRPL19* may also play a subtle role in basal resistance.

**Figure 3:**
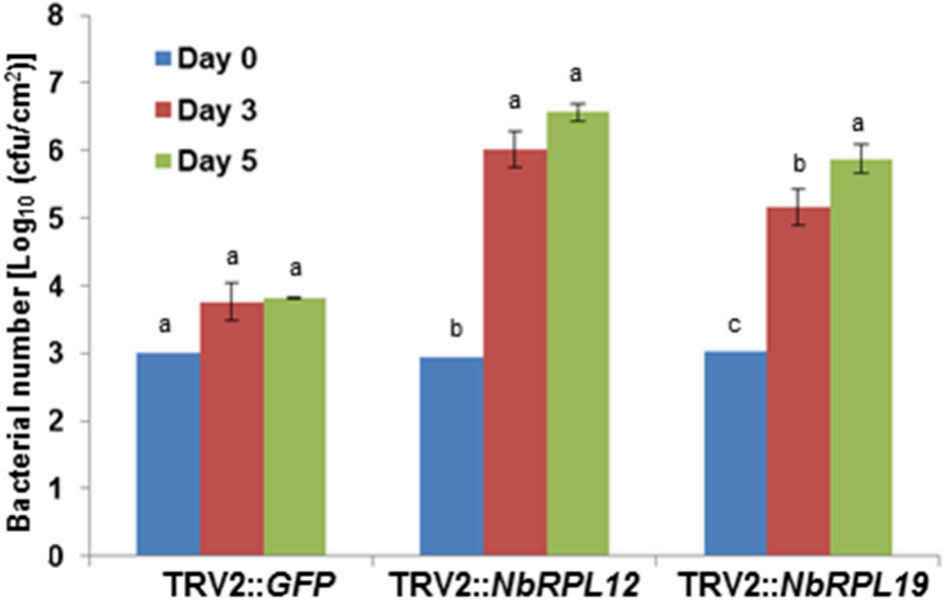
**Enhanced accumulation of nonhost pathogen ***P. syringae*** pv. tomato T1 on dip inoculated ***NbRPL12*** and ***NbRPL19*** gene silenced ***N. benthamiana*** leaves**. *NbRPL12* and *NbRPL19* silenced (as described for Figure 1) and control (TRV2::*GFP*) leaves were dip inoculated with *P. syringae* pv. tomato T1 (OD_600_ = 0.01 for 5 min). The bacterial population was quantified at 3 and 5 days post inoculation (DPI). Data was obtained from three biological replicates and the error bars represent standard deviation. Different letters above the bars indicate a significant difference from Two-way ANOVA at *p* < 0.05 with Tukey's HSD means separation test (α = 0.05) among different time points of each gene silenced plant. See Supplementary Table S2 for details about statistics.

As shown above, syringe infiltration of nonhost pathogens into *NbRPL12*- or *NBRPL19*-silenced plants suggested a role for these genes in apoplastic defense against host and nonhost pathogens. Further, *NbRPL12* and *NBRPL19*-silenced plants along with control plants were infected with nonhost pathogen, *P. syringae* pv. tomato T1 by dip inoculation. The bacterial multiplication in the plants was quantified at 3 and 5 dpi (Figure 3). At both these time points, the bacterial multiplication in *NbRPL12* and *NbRPL19* silenced plants were 10–100-fold higher as compared to the vector-only control plants (Figure 3). These results suggest that *NbRPL12* and *NbRPL19* silenced plants were compromised for nonhost resistance even when inoculated by dip inoculation method.

**Figure 4:**
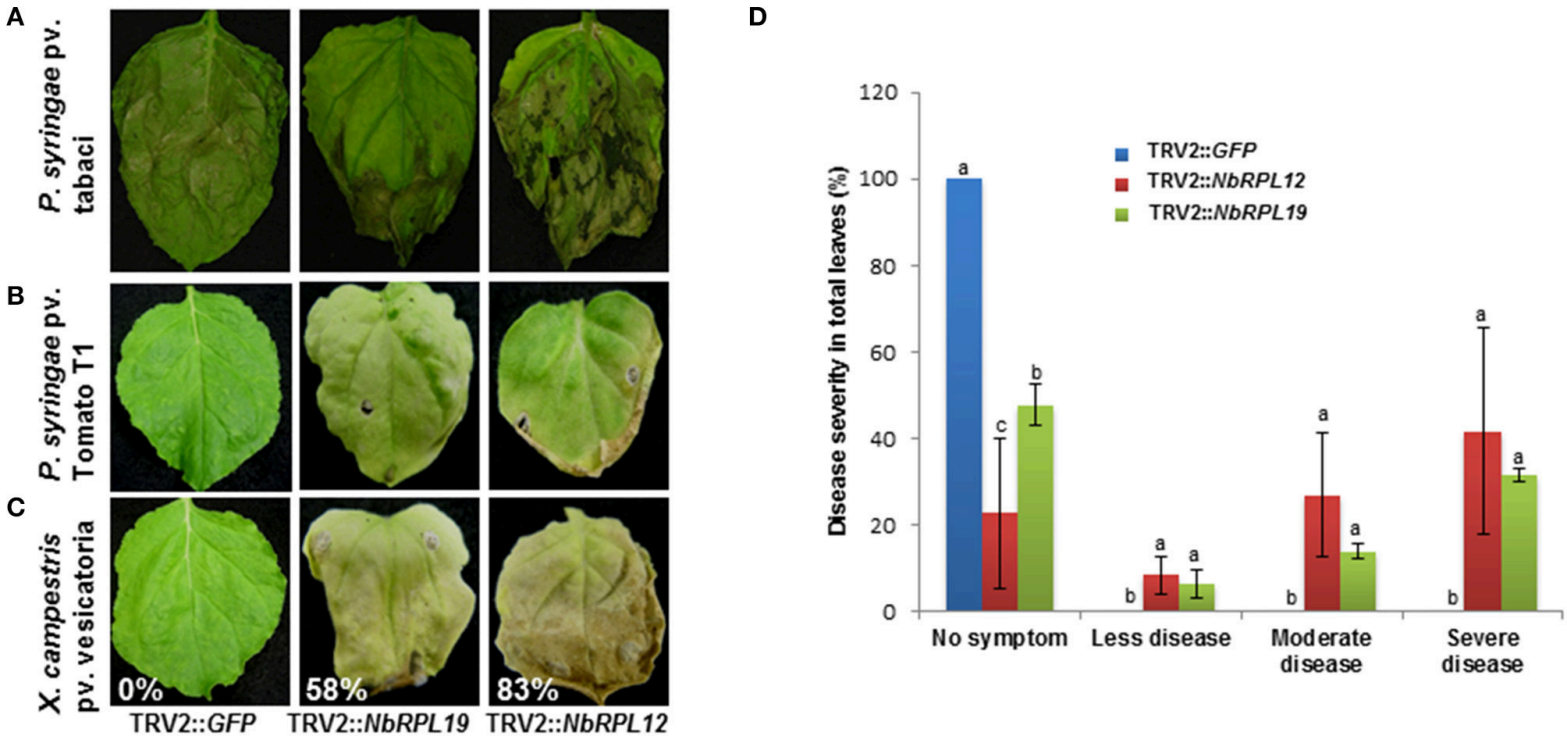
**Disease symptoms in the ***NbRPL12*** and ***NbRPL19*** gene silenced ***N. benthamiana*** plants inoculated with host and nonhost pathogens**. TRV2::*NbRPL12* or TRV2::*NbRPL19* inoculated *N. benthamiana* plants, along with control (TRV2::*GFP*), were individually inoculated by vacuum infiltration with either *P. syringae* pv. tabaci (**A**, OD_600_ = 0.0001) or *P. syringae* pv. tomato T1 (**B**, OD_600_ = 0.0002) or *X. campestris* pv. vesicatoria (**C**, OD_600_ = 0.0002) and the cell death phenotype was photographed 5 days **(A)** and 8 days **(B,C)** post inoculation. Percentage values denote number of leaves showing disease-induced visible cell death symptoms in one plant. Healthy leaves were scored as 0%. Bar graph denote the range of symptoms of *P. syringae* pv. tomato T1 diseased leaves in silenced and wild-type *N. benthamiana* plants **(D)**. The score was assigned by visually observing the leaves. Error bars represent standard deviation from three biological replicates. Different letters above the bars indicate a significant difference from Fisher's LSD and Bonferroni test for differences between means with Tukey's HSD means separation test (α = 0.05). See Supplementary Table S2 for details about statistics.

To investigate if the increased nonhost bacterial multiplication in the gene silenced plants causes disease symptoms, the silenced plants along with controls were infected with a host pathogen, *P. syringae* pv. tabaci, and nonhost pathogens *P. syringae* pv. tomato T1 and *X. campestris* pv. vesicatoria. The infected plants were monitored for disease progression (Figures 4A–C) and scored for disease symptoms (Figure 4D). There was a slight enhancement of disease symptoms with *NbRPL12* silenced plants when compared to vector control plants upon inoculation with the host pathogen *P. syringae* pv. tabaci and this was consistent with the bacterial multiplication when syringe infiltrated (Figure 2D). As expected, the vector control plants were resistant to nonhost pathogens and did not show any disease symptoms (Figures 4B,C). The host pathogen *Pseudomonas syringae* pv. tabaci caused disease symptoms on silenced plants and were similar to that of vector control plants (Supplementary Figure S4). Interestingly, both *NbRPL12* and *NbRPL19* silenced plants showed disease symptoms after inoculation with both nonhost pathogens tested. Severity of disease was scored based on the visual symptoms observed in Figure 4A and quantified based on an arbitrary scale of 0 to 4, 0 being no symptoms and 4 being severe symptoms (Figure 4D).

### *NbRPL12* and *NbRPL19* are induced upon pathogen inoculation

**Figure 5:**
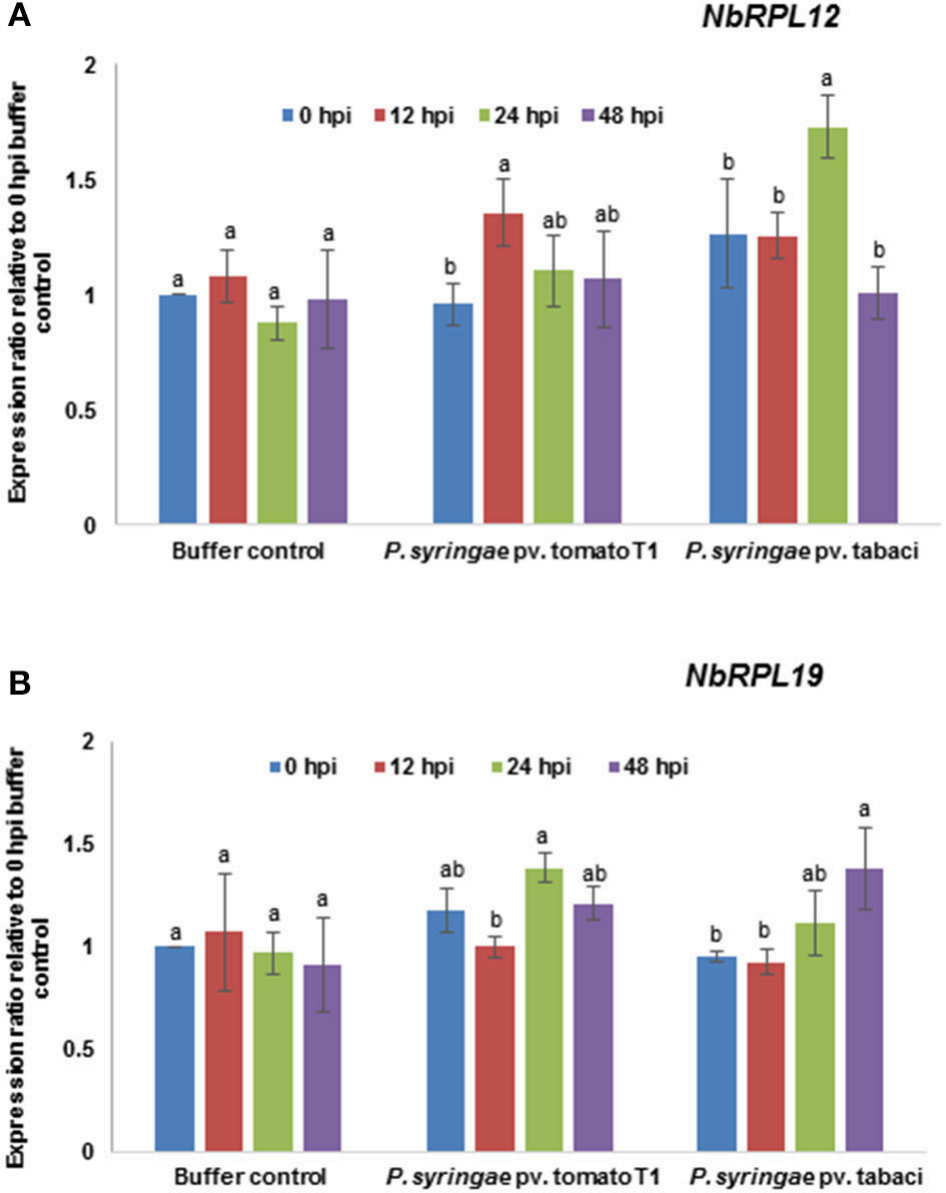
**Transcript expression pattern of ***NbRPL12*** and ***NbRPL19*** in wild-type ***N. benthamiana*** leaves challenged with host or nonhost pathogen**. *N. benthamiana* leaves were syringe inoculated with nonhost pathogen *P. syringae* pv. tomato T1 (*P. s*. tomato T1; OD_600_ = 0.0002) or host pathogen *P. syringae* pv. tabaci (*P. s*. tabaci; OD_600_ = 0.0001). The transcript levels of *NbRPL12* **(A)** and *NbRPL19* **(B)** genes were measured at indicated time points post inoculation by real-time quantitative RT-PCR. Data points are average of three biological replicates and error bars indicate the standard deviation. Different letters above the bar indicate a significant difference from Two-way ANOVA at *p* < 0.05 with Tukey's HSD means separation test (α = 0.05) among different time points within a treatment. Buffer control indicates leaves infiltrated with water. See Supplementary Table S2 for details about statistics.

The transcript levels of *NbRPL12* and *NbRPL19* upon host or nonhost pathogen inoculation were monitored by RT-qPCR (Figure 5). The data was compared with 0 hpi mock (buffer infiltrated) control. The *NbRPL12* gene was induced at 12 h post inoculation (hpi) in response to nonhost pathogen, *P. syringae* pv. tomato T1 and expression went down to buffer control level at 48 hpi (Figure 5A). *NbRPL12* was induced to 1.5-fold in response to the host pathogen *P. syringae* pv. tabaci at 24 hpi (Figure 5A). *NbRPL19* gene was induced only during host pathogen inoculation at 48 hpi (Figure 5B).

### Double silencing of *NBRPL12* and *NbRPL19* does not have an additive phenotype

As mentioned earlier, both *NbRPL12* and *NbRPL19* silenced plants showed a delay in HR upon inoculation with nonhost pathogens. However, the delay of HR in *NbRPL12* silenced plants was stronger when compared to *NbRPL19* silenced plants (Figure 1). To further characterize this difference in the delay of HR and to determine if these two genes have an additive effect on nonhost resistance, double silencing was performed and monitored for HR. Prior to assaying for delay in HR, the transcript levels of *NbRPL12* and *NbRPL19* in the double-silenced plants were monitored. Transcripts of both the genes were significantly less when compared to non-silenced vector control plants (Supplementary Figure S5).

**Figure 6:**
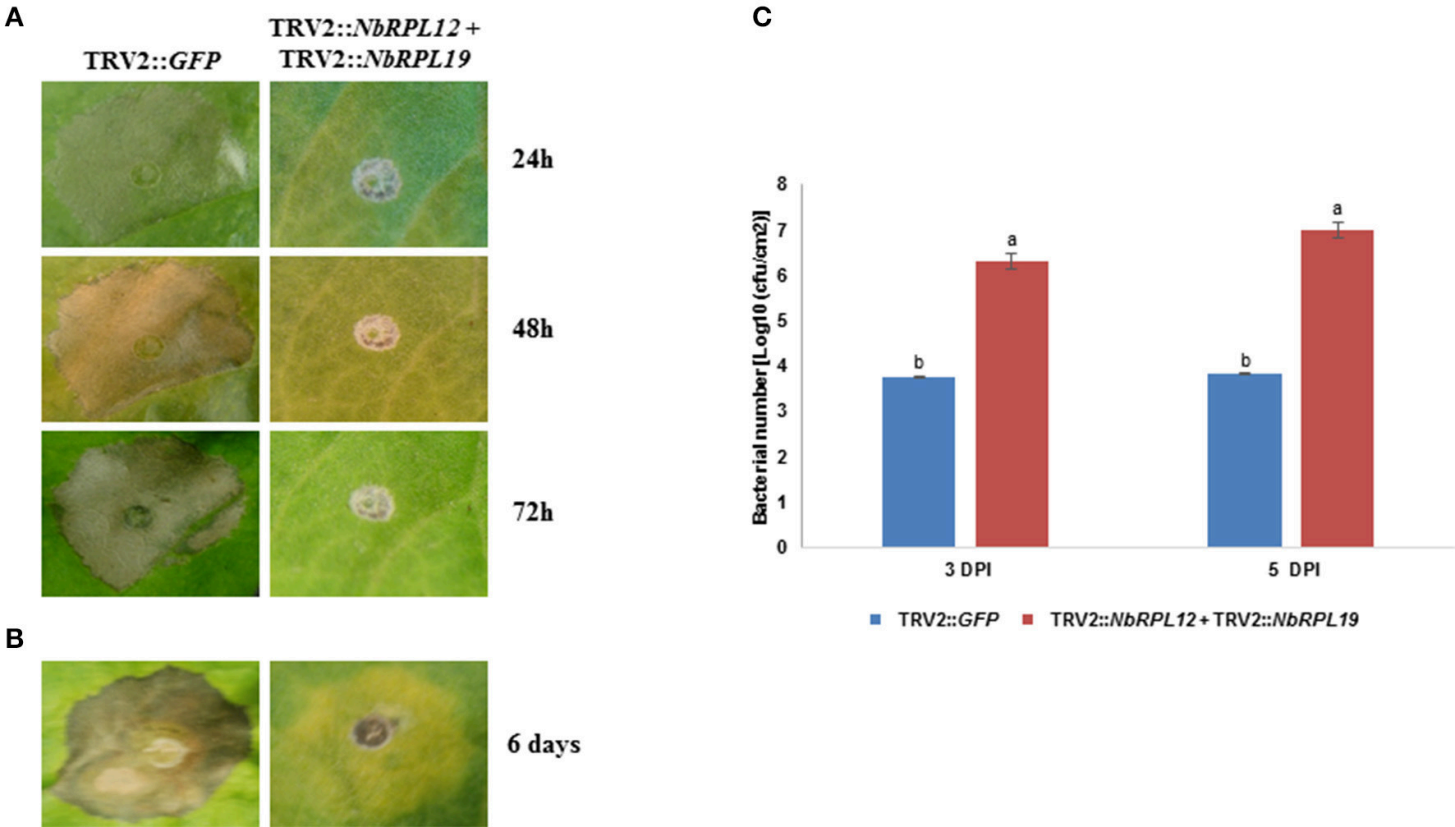
**Double-silenced plants showed delayed HR and enhanced bacterial multiplication upon inoculation with nonhost pathogens**. The silenced leaves were inoculated using a needless syringe with nonhost pathogens *P. syringae* pv. tomato T1 (**A**, OD_600_ = 0.001) and *X. campestris* pv. vesicatoria (**B**, OD_600_ = 0.01). The delay in HR symptoms was photographed at the indicated time points post inoculation. Silenced leaves were dip inoculated with *P. syringae* pv. tomato T1 (OD600 = 0.01 for 5 min). The growth response of pathogen was quantified at 3 and 5 days post inoculation (DPI) **(C)**. The error bars represent standard deviation from three replications. Different letters above the bar indicate a significant difference from Two-way ANOVA at *p* < 0.05 with Tukey's HSD means separation test (α = 0.05). See Supplementary Table S2 for details about statistics.

Occurrence of HR was delayed in the double-silenced (*NbRPL12* + *NbRPL19*) plants against *P. syringae* pv. tomato T1 (Figure 6A) and *X. campestris* pv. vesicatoria (Figure 6B). Further, *NbRPL12* + *NbRPL19* double-silenced plants were infected with nonhost pathogen *P. syringae* pv. tomato T1 and the bacterial multiplication was quantified. Corresponding to the delay in HR, double-silenced plants supported more bacterial multiplication as compared to vector control (Figure 6C). Based on the bacterial count, it can be concluded that the double-gene silencing also compromises nonhost disease resistance but the bacterial multiplication was not significantly more than single gene silenced plants.

### Loss of *RPL12* and *RPL19* gene expression in *Arabidopsis* allowed multiplication of nonhost pathogen

**Figure 7:**
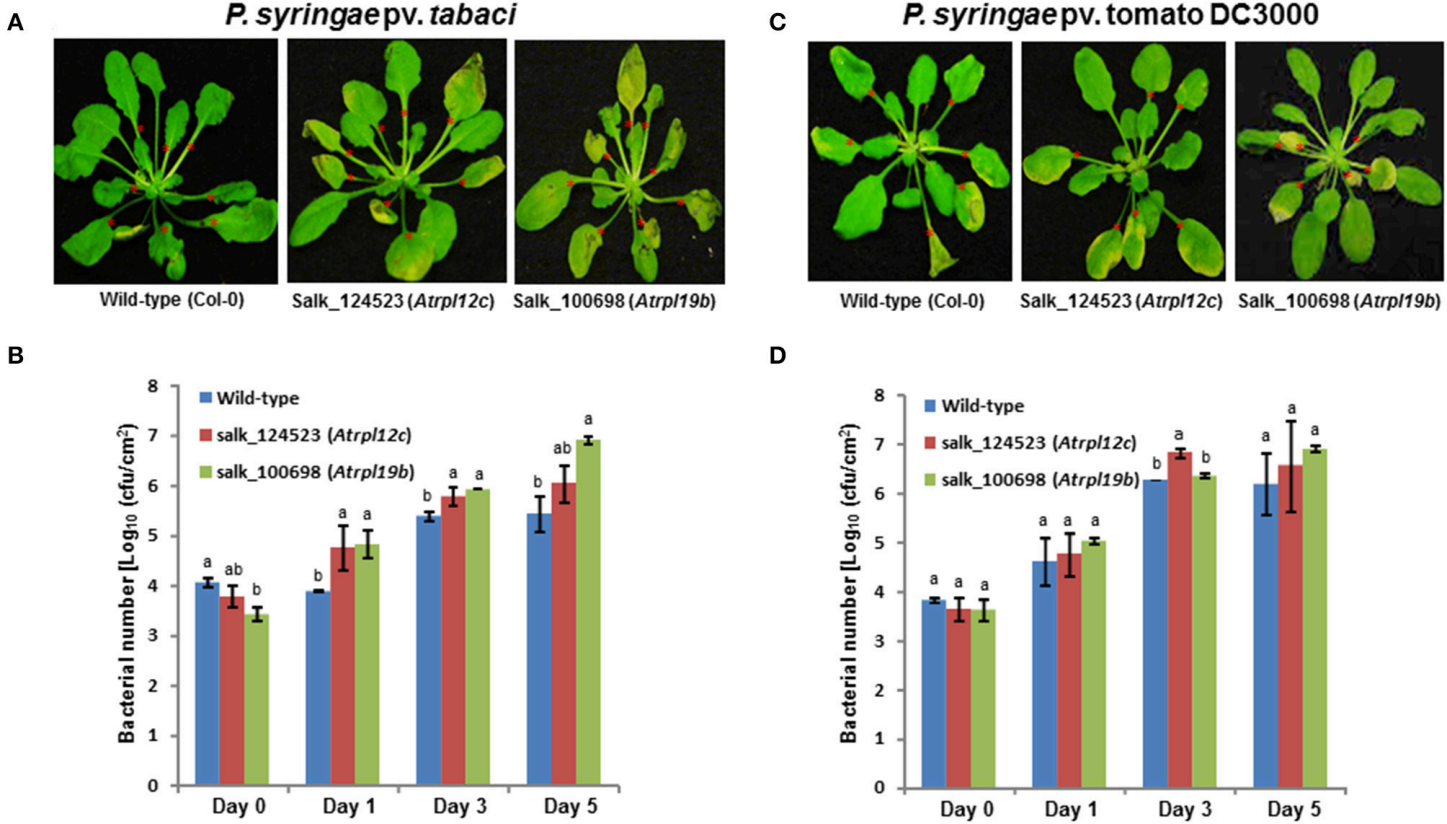
**Multiplication of nonhost pathogen and host pathogen on ***Atrpl12*** and ***Atrpl19*** mutants of ***Arabidopsis*****. The T-DNA knock-out mutants of *Atrpl12* and *Atrpl19* were inoculated with *P. syringae* pv. tabaci (**A,B**, OD_600_ = 0.001) or *P. syringae* pv. tomato DC3000 (**C,D**, OD_600_ = 0.0001) using needleless syringe. The phenotype of nonhost or host pathogen inoculated plants were photographed at 4 days post inoculation (dpi; **A**) or 5 dpi **(C)** and bacterial multiplication was quantified at 0, 1, 3, and 5 dpi **(B,D)**. An average of 7 leaves per plant were inoculated (indicated with red asterisks). Data was taken from three biological replicates and the error bars represent standard deviation. Different letters above the bar indicate a significant difference from Two-way ANOVA at *p* < 0.05 with Tukey's HSD means separation test (α = 0.05) among the genotypes within a time point. See Supplementary Table S2 for details about statistics.

To assess if *RPL12* and *RPL19* have a role in nonhost resistance in another plant species, individual Arabidopsis knockout mutants of Salk_124523 for *RPL12C* (At5g60670) and Salk_100698 for *RPL19B* (At3g16780) genes were identified (Supplementary Figure S6) in the SALK database and were obtained from Arabidopsis biological resource center. Homozygous mutants were selected and were infected with either a nonhost pathogen (*P. syringae* pv. tabaci) or a host pathogen (*P. syringae* pv. tomato DC3000). Consistent with the results obtained in *N. benthamiana*, disruption of *AtRPL12* or *AtRPL19* expression caused disease in the mutant plants when infected with the nonhost pathogen, *P. syringae* pv. tabaci (Figure 7A). In contrary, the wild-type (Col-0) plants did not show any disease symptoms upon inoculation with *P. syringae* pv. tabaci. Congruent to the disease progression, an increase in bacterial number in the mutants, when compared to wild-type Col-0, was observed for the nonhost pathogen tested (Figure 7B).

Similar to that of results from *N. benthamiana* silenced plants, the Arabidopsis *rpl19* mutant did not show any significant difference in disease progression in contrast to wild-type Col-0 upon inoculation with the host pathogen, *P. syringae* pv. tomato DC3000. However, the *rpl12* mutant showed slight increase in bacterial multiplication when compared to Col-0 at 3 dpi (Figures 7C,D).

## Discussion

Bacterial pathogens have the potential to evade plant defense and invade plant apoplastic space and draw nutrients from the host plant cell (Jones and Dangl, 2006; Wang et al., 2012). Later, these bacteria multiply, form colonies and eventually cause disease. Host pathogens can hijack the host cellular machinery to cause disease (Jones and Dangl, 2006). In contrary, nonhost pathogens cannot infect the plant due to the existence of a broad spectrum plant defense mechanism called nonhost resistance (Heath, 2000; Mysore and Ryu, 2004). HR-mediated defense response contributes to prevent further growth of nonhost bacterial pathogens that eventually leads to resistance.

We have used VIGS-based forward genetics screen to identify genes contributing to nonhost resistance in *N. benthamiana* (Rojas et al., 2012). From this screen we identified that silencing of ribosomal genes, *NbRPL12* and *NbRPL19*, compromised *P. syringae* pv. tomato T1 induced HR. In this manuscript, we further characterized these genes and showed the involvement of RPL12 and RPL19 proteins in production of HR cell death and apoplastic growth of bacteria. Consistent with our results, several other studies have identified ribosomal proteins, in forward genetic screens, to play a role in HR (Lu et al., 2003; Gabriëls et al., 2006). However, the role of these proteins in plant defense was not further characterized.

RPL12 and RPL19 proteins in different species are known to be involved in proper functioning of a number of factors involved in ribosome biogenesis and protein synthesis in ribosomes (Plafker and Macara, 2002). RPL12 and RPL19 proteins are known to be targeted to nucleolus following their synthesis in cytoplasm. In contrast, nuclear genome encoded RPLs are known to be localized to chloroplast in rice (Kusaka et al., 1998) and Arabidopsis (Weglöehner and Subramanian, 1994). RPLs are shown to be localized to mitochondria in potato (Delage et al., 2007).

Apart from its role in translation machinery, RPL proteins are known to play vital extra-ribosomal roles such as the involvement in plant-pathogen interactions (Gabriëls et al., 2006; Yang et al., 2009). For example, *RPL19, RPL13* and *RPL7* genes were attributed to play a role in *Turnip mosaic virus* and *Tobacco mosaic virus* accumulation in *N. benthamiana* (Yang et al., 2009). *HLL* (HUELLENLOS) gene, that encodes RPL14, has been demonstrated to be involved in patterning and growth of the Arabidopsis ovule (Skinner et al., 2001).

Both *NbRPL12* and *NbRPL19* gene silenced *N. benthamiana* plants showed varying extent of delay in initiation of HR against nonhost pathogens *X. campestris* pv. vesicatoria and *P. syringae* pv. tomato T1. This delayed defense response may have contributed to compromising the nonhost resistance against these bacteria and hence they could grow to a certain extent more in the silenced plants when compared to non-silenced control plants. Interestingly, these nonhost pathogens were also able to cause disease symptoms in the silenced plants indicating that the gene silenced plants compromised a range of defense responses. An earlier study has also shown that Cf4-Avr4 induced HR was delayed in *N. benthamiana* plants silenced for *NbRPL19* (Gabriëls et al., 2006). Similarly, another forward genetics screen showed that 22 out of 79 genes suppressing Pto-AvrPto induced HR were various ribosomal proteins (Lu et al., 2003). Therefore, the specific involvement of ribosomal proteins in plant defense response depends on how the defense response is initiated. For example NbRPL12 and NbRPL19 may be specifically involved when the defense response is initiated against nonhost pathogens while other ribosomal proteins maybe involved during defense response initiated by a specific gene-for-gene interaction.

One possible explanation for the role of *RPL12* and *RPL19* in plant defense can be its activation and subsequent involvement in *de novo* synthesis of certain proteins that play a role in plant defense. For example, in mouse erythroblasts, downregulation of *RPL19* and *RPL11* show specific drop in translation of 130 mRNAs that are responsible for differentiation of erythroid precursor (Horos et al., 2012). Consistent with our hypothesis, for example, *eIF4A* (a translation initiation factor) gene silenced plants in our forward genetics screen also compromised HR against nonhost pathogen, *P. syringae* pv. tomato T1 (Rojas et al., 2012). Apart from susceptibility to type-II nonhost pathogens that cause visible HR (Mysore and Ryu, 2004), the *NbRPL12* and *NbRPL19* silenced plants were also partially susceptible to *P. syringae* pv. glycinea, a type-I nonhost pathogen that doesn't cause any visible symptoms (Mysore and Ryu, 2004). In Arabidopsis, RPL12 interacting receptor for activated C- kinase (RACK1) was identified (Kundu et al., 2013). RACK1 plays a key role in innate immunity as part of a regulatory protein complex (Nakashima et al., 2008; Wang et al., 2014). All together these data suggest that RPL12 and RPL19 play a role not only in basal defense responses but also in nonhost resistance.

Generally after a pathogen invades preformed constitutive and inducible barriers, it will be subject to recognition at the plasma membrane. At this point, PAMPs can be recognized and plants induce PTI (Jones and Dangl, 2006). PTI induces expression of several defense related genes. *RPL12* and *RPL19* were also induced in response to host and nonhost pathogens. We therefore speculate that PAMPs may induce *RPL12* and *RPL19*. Consistent with our data, transcripts of several genes encoding ribosomal proteins were shown to be altered in Arabidopsis plants expressing *Pti4* that encodes an ethylene-responsive factor (Chakravarthy et al., 2003). In addition, a previous study has shown that silencing of *RPL19* gene in *N. benthamiana* delays HR induced by a PAMP, Inf1 (Gabriëls et al., 2006), indicating that *RPL19* may be involved in PAMP-mediated defense pathway.

In addition to *N. benthamiana*, we also show that *RPL12* and *RPL19* may have a role in nonhost resistance in Arabidopsis. It is to be noted that we were able to test only one allelic mutant for *AtRPL12* and *AtRPL19* genes. Even though the role of *AtRPL12* and *AtRPL19* in Arabidopsis nonhost resistance is not proven, the fact that both *rpl12* and *rpl19* mutants compromised nonhost resistance suggest that *RPLs* may play a role in nonhost disease resistance in different plant genera. Since both *AtRPL12* and *AtRPL19* are members of a gene family, it is intriguing that mutation in just one member of the gene family shows partial loss of nonhost resistance phenotype. It could be due to a dosage effect and it is possible that when more than one member of the gene family is knocked out we could see an additive effect with regards to growth of nonhost bacteria. Alternatively, the differential expression of genes within a family during various stresses can contribute to non-redundant function of the gene family members. Based on the publicly available gene expression data in Geneinvestigator (https://genevestigator.com/gv/doc/intro_plant.jsp) we know that members of *AtRPL12* and *AtRPL19* are differentially expressed in response to various stresses. The precise role of *AtRPL12* and *AtRPL19* in plant defense needs further investigation. Nevertheless, taken together, our data implicates a role for plant *RPL12* and *RPL19* genes in nonhost resistance against bacterial pathogens.

## Materials and methods

### Plant growth

*N. benthamiana* seeds were germinated and grown in green house conditions as described earlier (Wang et al., 2012). Arabidopsis T-DNA lines (Salk_124523, AtRPL12 and Salk_100698, AtRPL19) were obtained from Arabidopsis biological resource center (ABRC). The seeds were germinated in pots after cold treatment for 3–4 days at 4°C as described earlier (Rojas et al., 2012; Senthil-Kumar et al., 2013). The soil used for all plant growth was from SUNGRO Horticulture Distribution Inc. Bellevue, WA. Silenced *N. benthamiana* plants, 3–4 weeks post TRV inoculation, or 4–5 weeks old Arabidopsis plants were used for all the assays.

### VIGS

*Agrobacterium tumefaciens* GV2260 containing either pTRV1 or pTRV2 vector (Liu et al., 2002) with *NbRPL12* or *NbRPL19* gene fragment (Supplementary Figure S7) and were grown overnight at 28° C in Luria-Bertani (LB) medium containing rifampicin at 25 μg/ml and kanamycin (both from Sigma. St. Louis, MO) at 100 μg/ml. Cells were harvested, resuspended in induction medium containing 10% Mannitol, 30 mM MES, pH 5.5 and acetosyringone at a concentration of 200 nm/ml and incubated at room temperature for 4–5 h with slow shaking. Following induction, cells were harvested, resuspended in infiltration medium (10 mM MES, pH 5.5), the optical density (OD) at 600 nm adjusted to 0.8 for both pTRV1- and pTRV2 containing Agrobacteria, mixed at 1:1 ratio and infiltrated using needle-less syringe into the lower leaves of 3 week old *N. benthamiana* plants (Senthil-Kumar et al., 2013; Senthil-Kumar and Mysore, 2014). We used a software (http://bioinfo2.noble.org/RNAiScan.htm; Xu et al., 2006) to find off-targets for the gene fragments cloned in the construct. For both the genes, no off-targets were predicted.

### Pathogen inoculation

*P. syringae* pv. tomato TI, *P. syringae* pv. tabaci, *P. syringae* pv. glycinea and *X. campestris* pv. vesicatoria were grown overnight at 28°C in King's B medium containing appropriate antibiotics. The cells were harvested by centrifugation at 5000 rpm for 10 min followed by resuspension in 10 mM MgCl_2_. The OD at 600 nm was monitored; cells were diluted to the required OD in 10 mM MgCl_2_ with 0.01% (v/v) Silwet L-77 (Osi Specialties, Friendship, WV). The diluted pathogen culture was infiltrated into the plants using a needle-less syringe or by vacuum infiltration or dip inoculation. Bacterial multiplication was calculated as described earlier (Rojas et al., 2012).

### Disease scoring

*N. benthamiana* plants were inoculated with pathogens and at 8 dpi the disease symptoms were quantified by visual scoring. Necrotic area in the pathogen inoculated leaves was visually scored as 0 (no disease cell death) to 4 (severe disease cell death) and expressed as percentage values.

### HR and chemical-induced cell death assay

*N. benthamiana* plants were inoculated with indicated concentrations of nonhost pathogen and the HR symptoms were observed daily up to 6 dpi. In order to know the changes in gene-for-gene HR we used fungal and bacterial *R*-*Avr* gene product interactions as follows. Leaves were infiltrated with a mixture of *Agrobacterium* carrying either *Pro35S:tvEIX* + *Pro35S:LeEix2* or *Pro35S:AvrPto* + *Pro35S:Pto* or *Pro35S:Avr9* + *Pro35S:Cf9*. Plants were maintained under standard growth conditions until the observation of cell death. The cell death phenotype was photographed 10 days post inoculation. In order to assess whether the cell death response is a generic, but not programmed like HR, we independently inoculated the plants with ethanol, NaCl and H_2_O_2_. Cell death symptoms caused by these chemicals were photographed as indicated in figure legends.

### Quantitative RT-PCR (RT-qPCR)

Endogenous transcript levels in gene-silenced plants were quantified using RT-qPCR. The total RNA was extracted from silenced and mock infiltrated plants and the first-strand cDNA was synthesized using oligo (dT)_15_ primers. RT-qPCR was performed using ABI PRISM 7000 (ABI applied biosystems Inc., Foster city, CA) using SYBR green (ABI). The primers used were designed using primer quest software (Integrated DNA Technologies, Inc, Coralville, Iowa USA). Parallel reaction using *N. benthamiana* Elongation factor 1-α (*EF1*) was performed and the data obtained were used to normalize respective gene transcripts. Each sample was run in triplicate from pooled samples. Endogenous transcript levels of respective genes were calculated by following the protocol as described earlier (Pfaffl, 2001). Primer details are given in Supplementary Table S1, Supplementary Figures S8, S9.

## Author contributions

MS, KW, VR, and SN designed the experiments, generated the required materials for all experiments and performed the experiments. SN, MS, and KM wrote the manuscript. All the authors have read and agreed with the submission of manuscript.

### Conflict of interest statement

The authors declare that the research was conducted in the absence of any commercial or financial relationships that could be construed as a potential conflict of interest.
