# Supplementary figures and images for: Plant Ribosomal Proteins, RPL12 and RPL19, Play a Role in Nonhost Disease Resistance against Bacterial Pathogens
Source: Front Plant Sci. 2016 Jan 6;6:1192. doi: 10.3389/fpls.2015.01192 (PMC4702080; doi:10.3389/fpls.2015.01192)

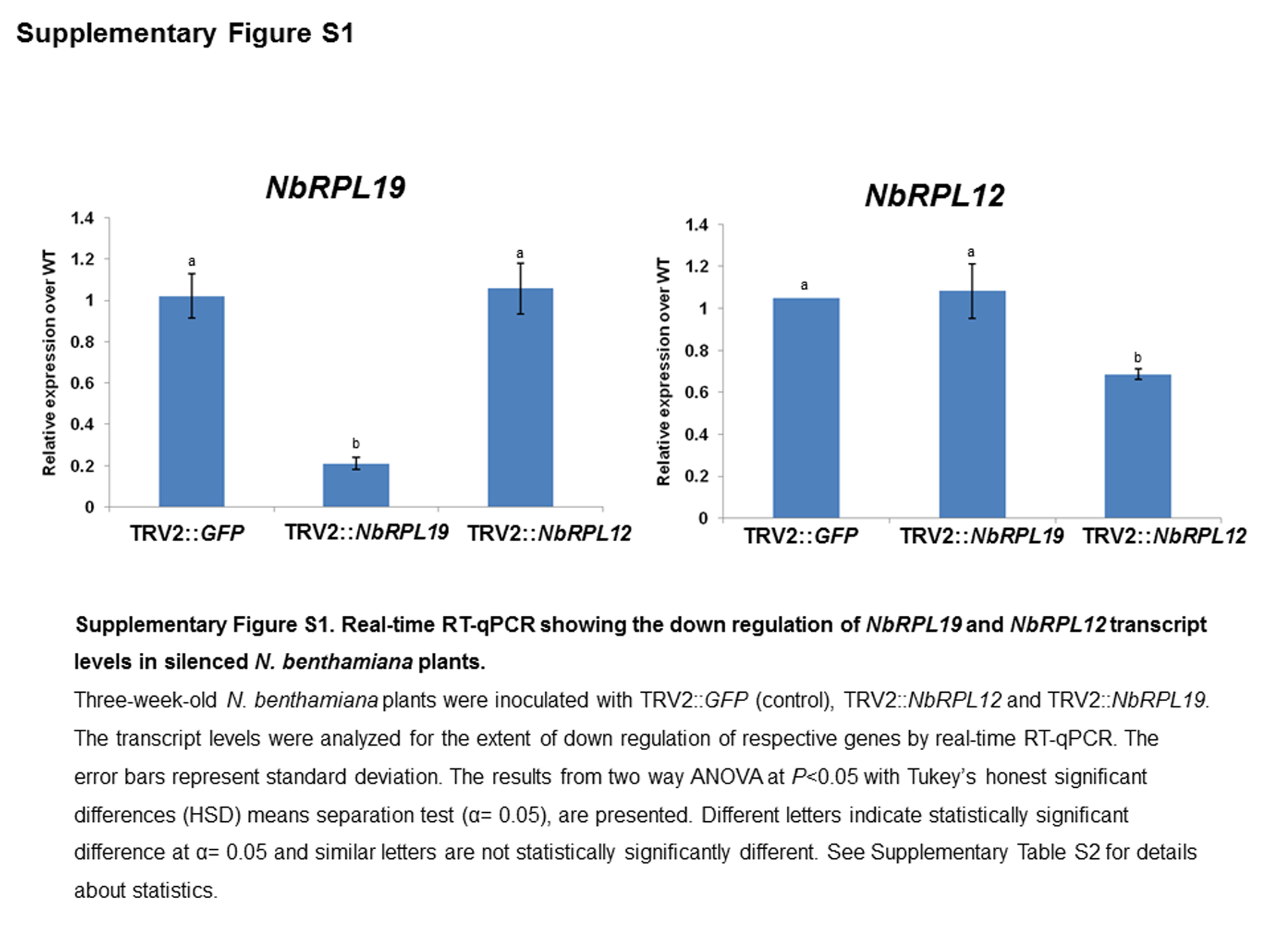

Supplement: Supplementary file 1 [file Image1.tif]

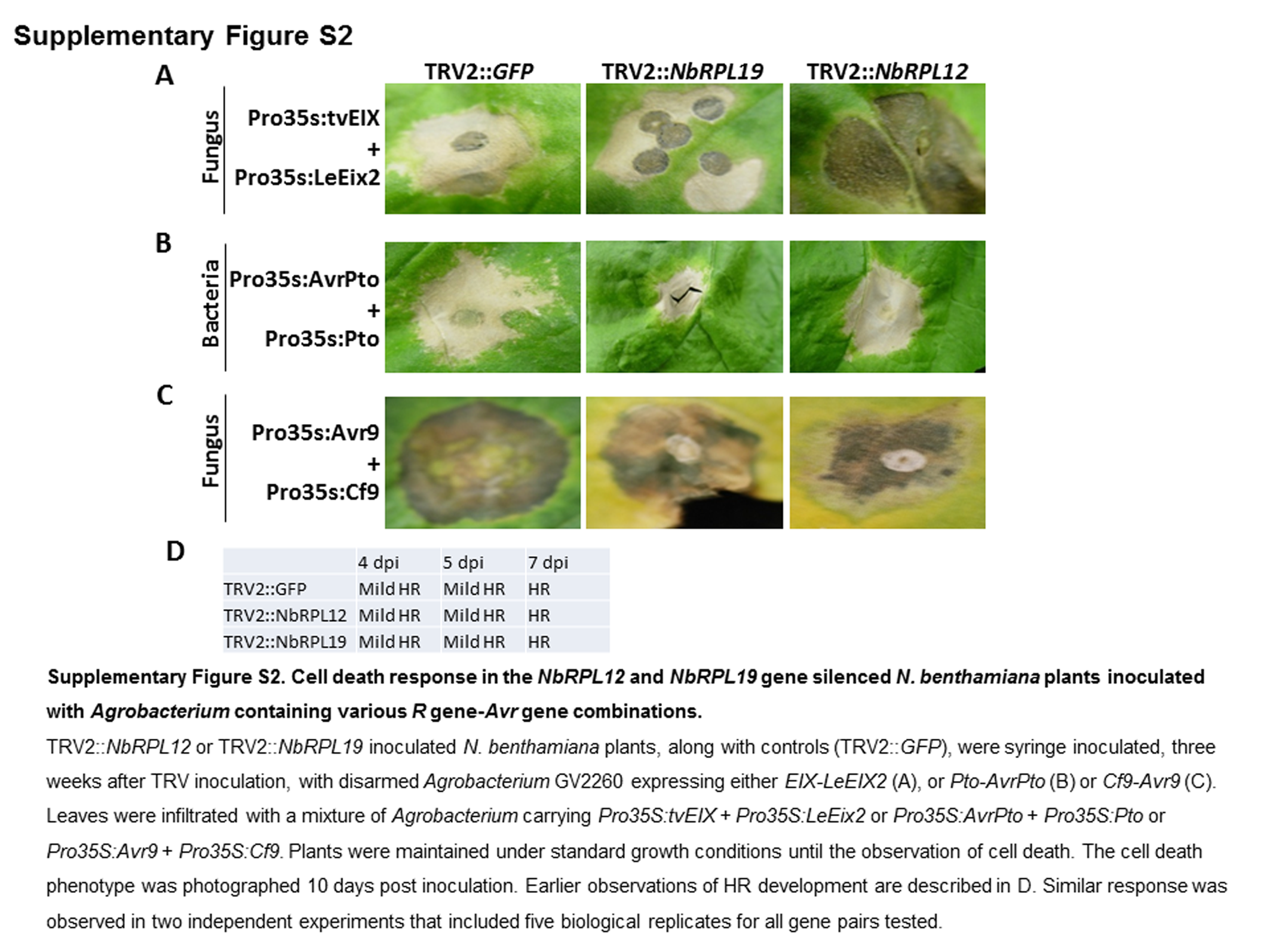

Supplement: Supplementary file 2 [file Image2.TIF]

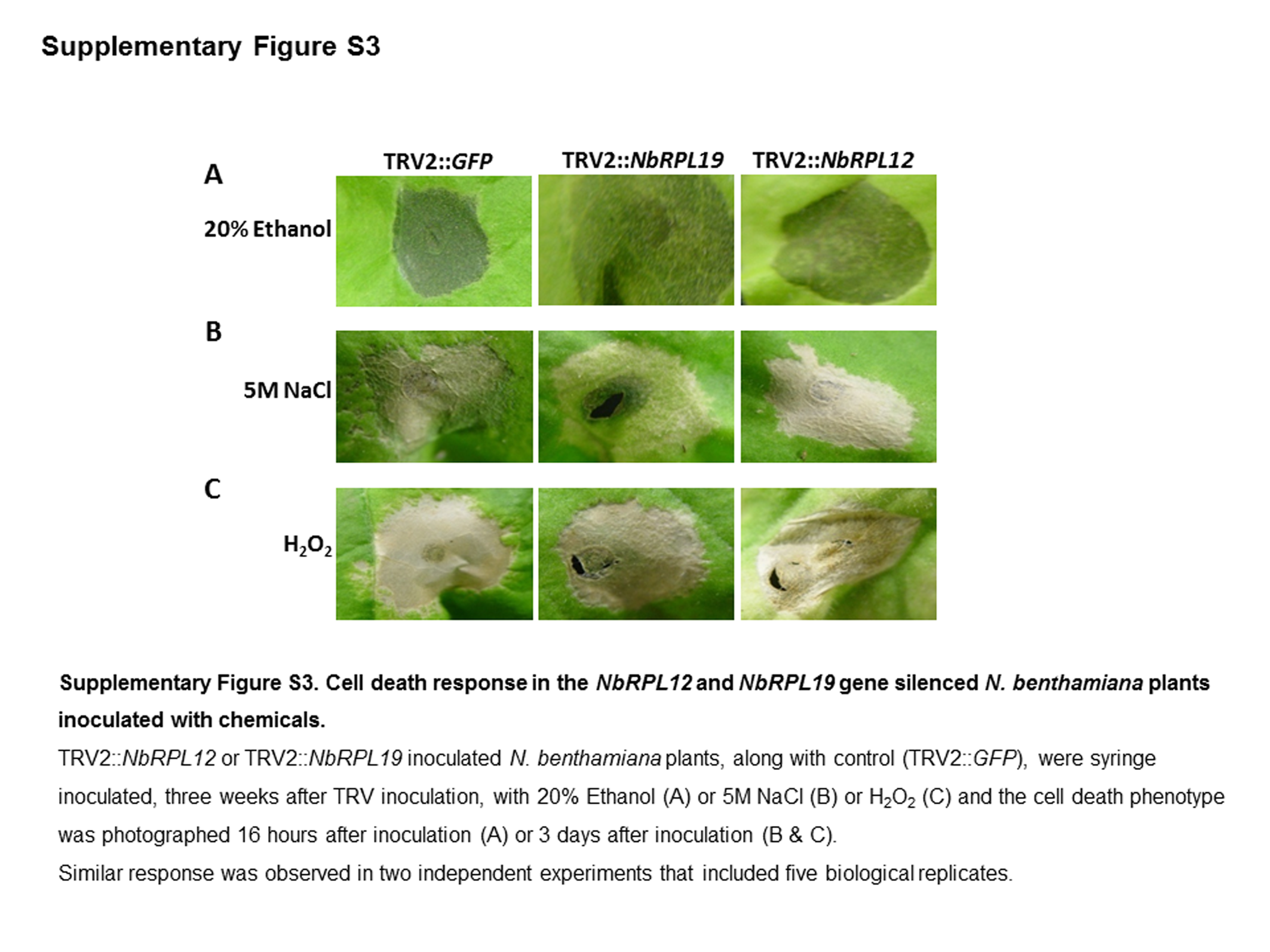

Supplement: Supplementary file 3 [file Image3.TIF]

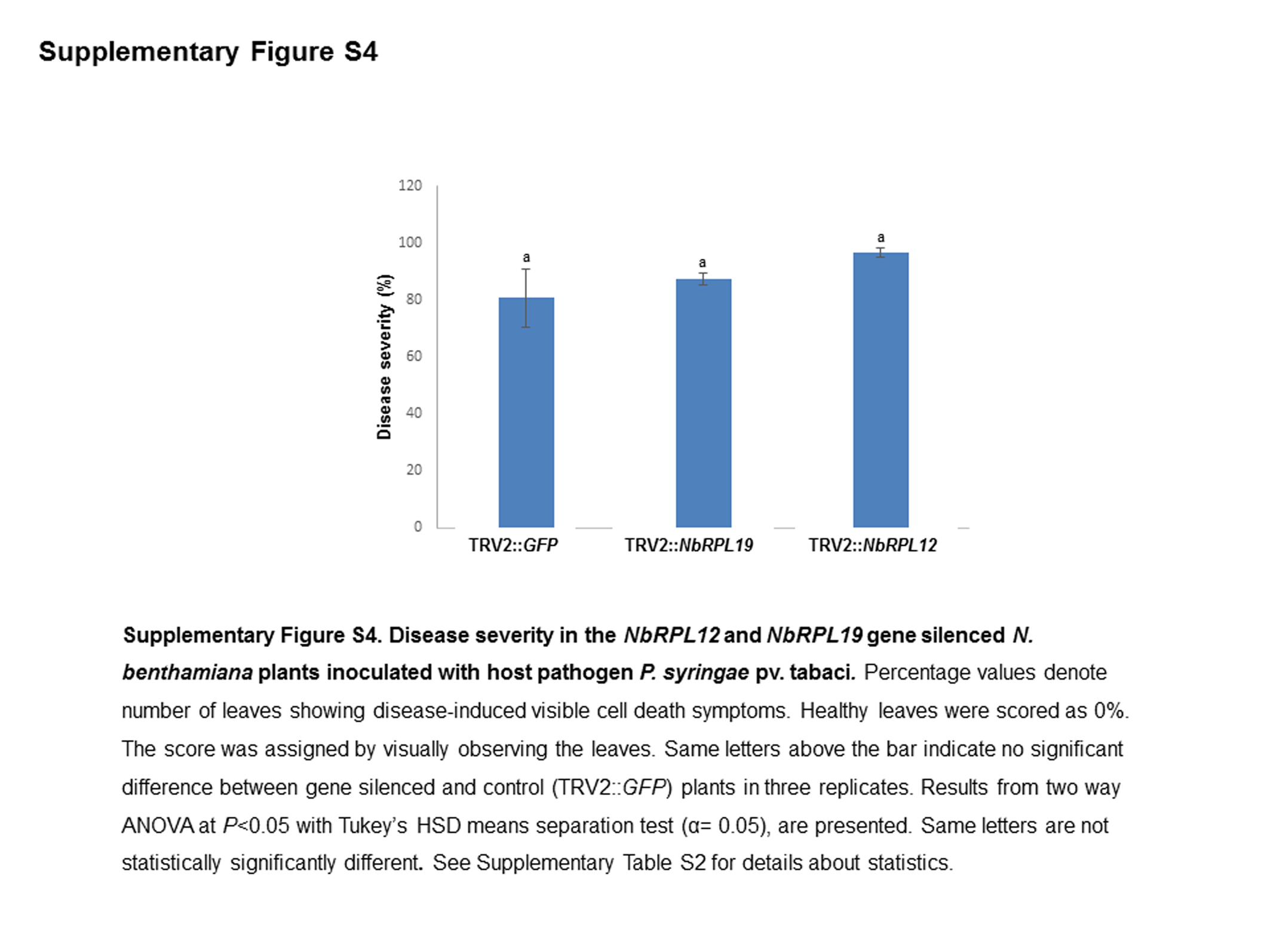

Supplement: Supplementary file 4 [file Image4.tif]

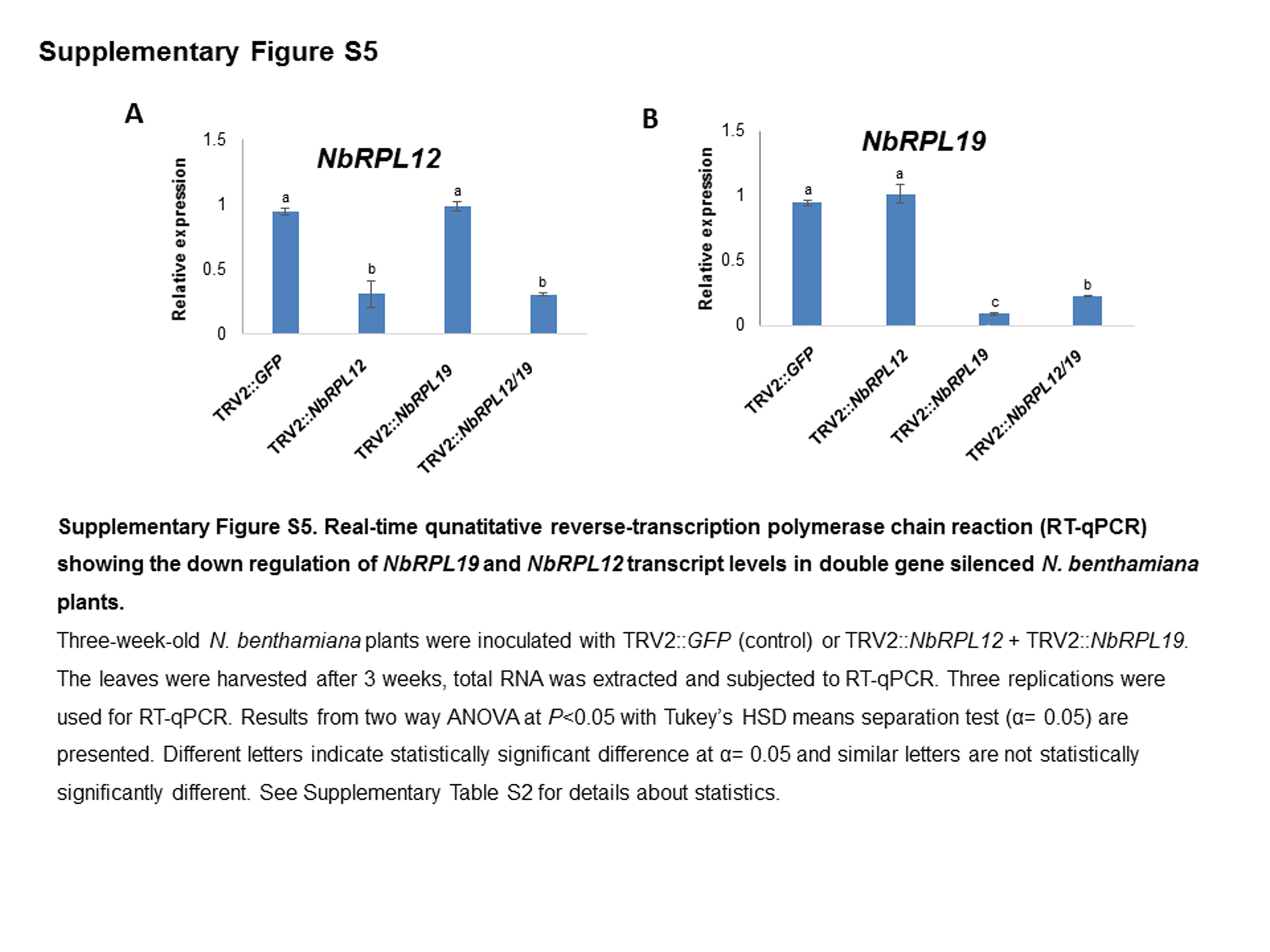

Supplement: Supplementary file 5 [file Image5.tif]

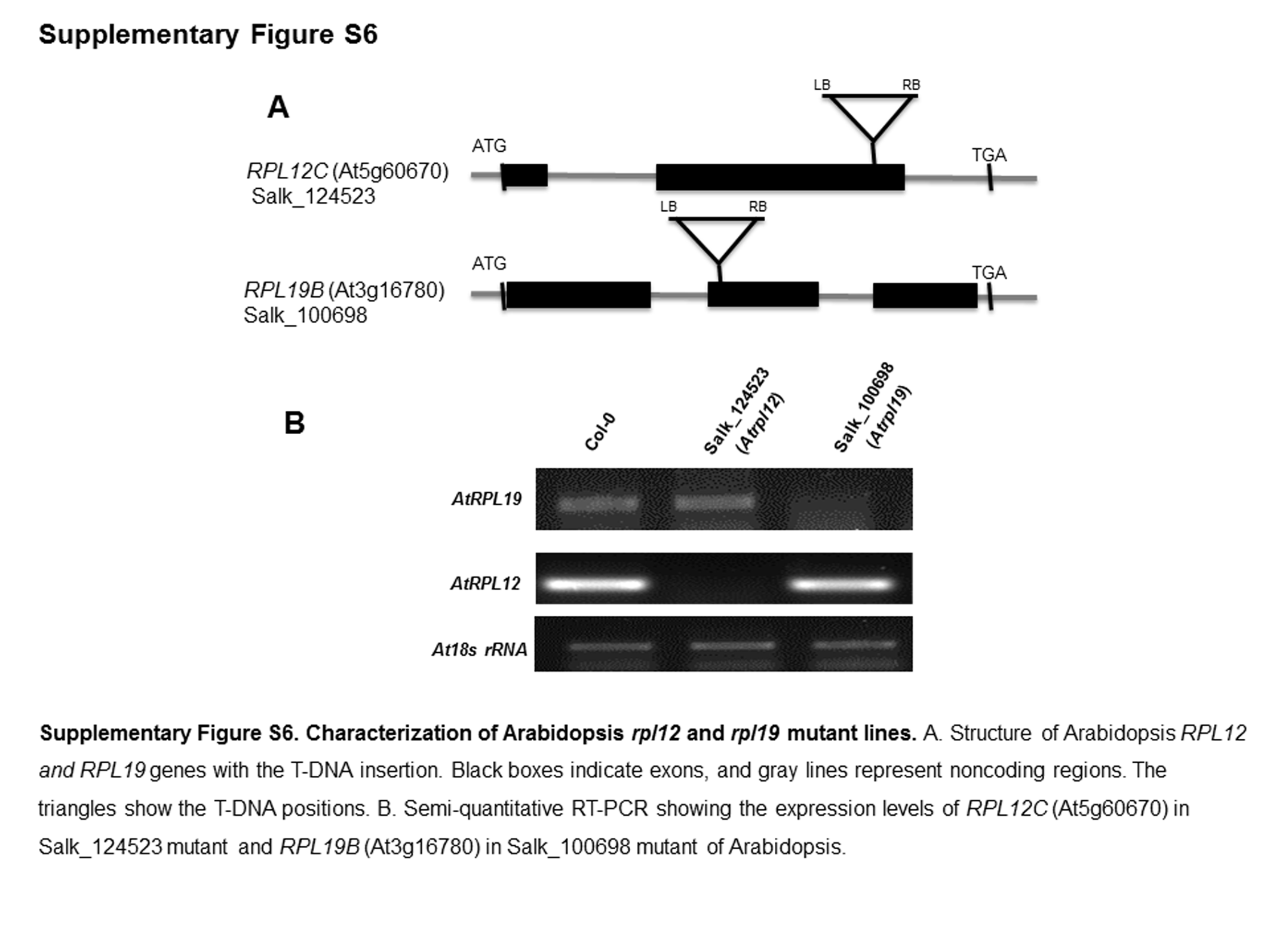

Supplement: Supplementary file 6 [file Image6.TIF]

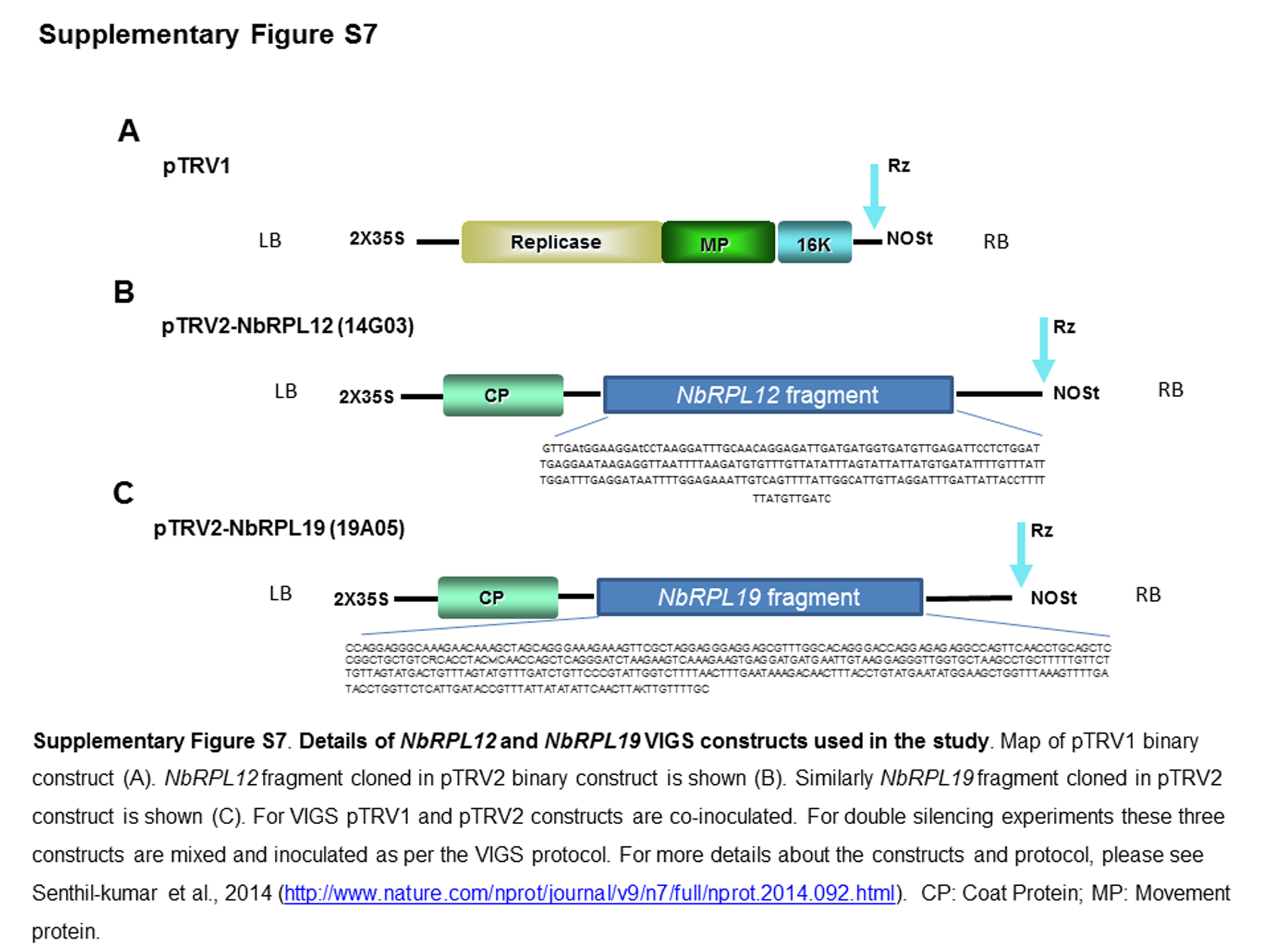

Supplement: Supplementary file 7 [file Image7.TIF]

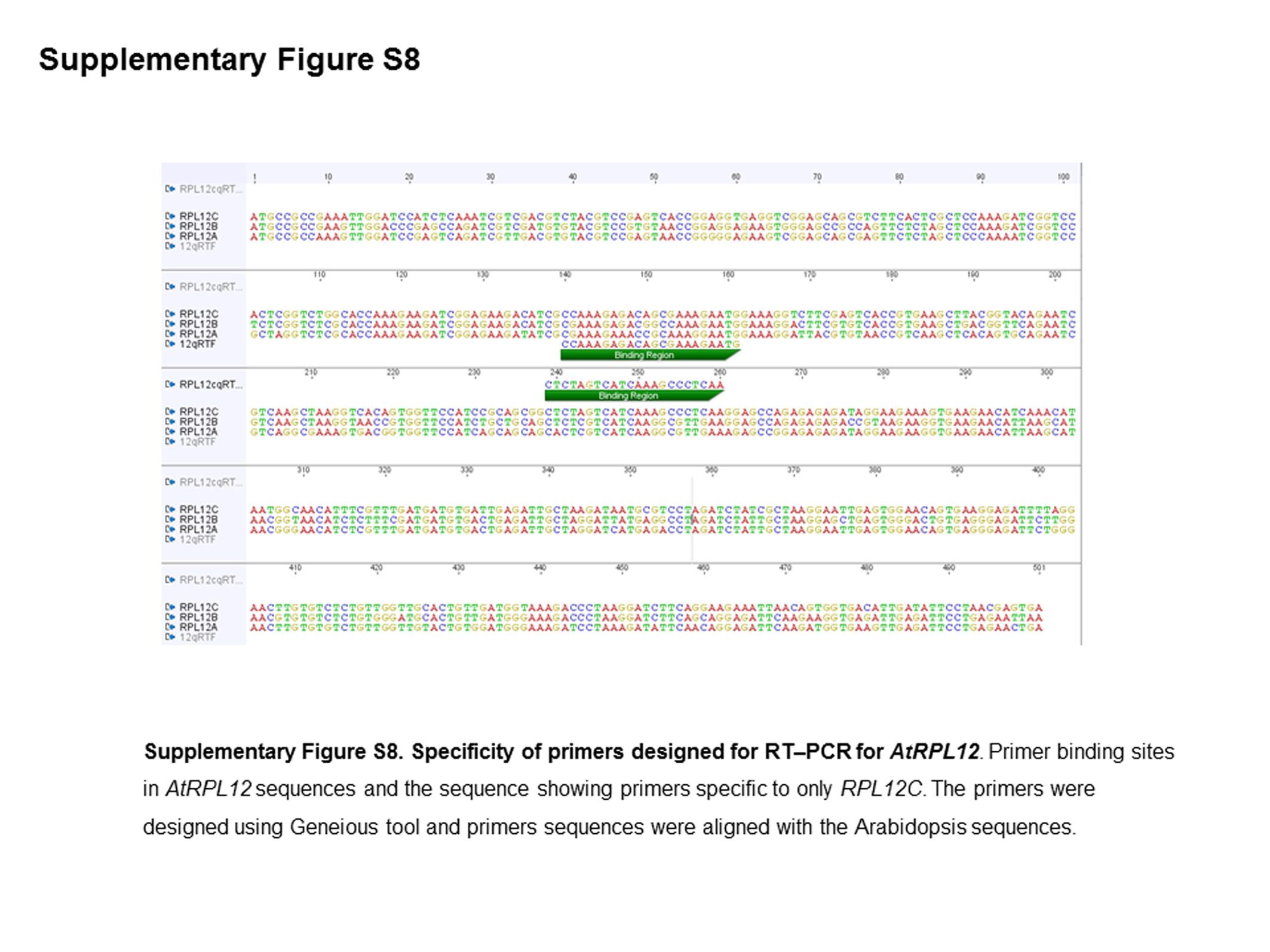

Supplement: Supplementary file 8 [file Image8.TIF]

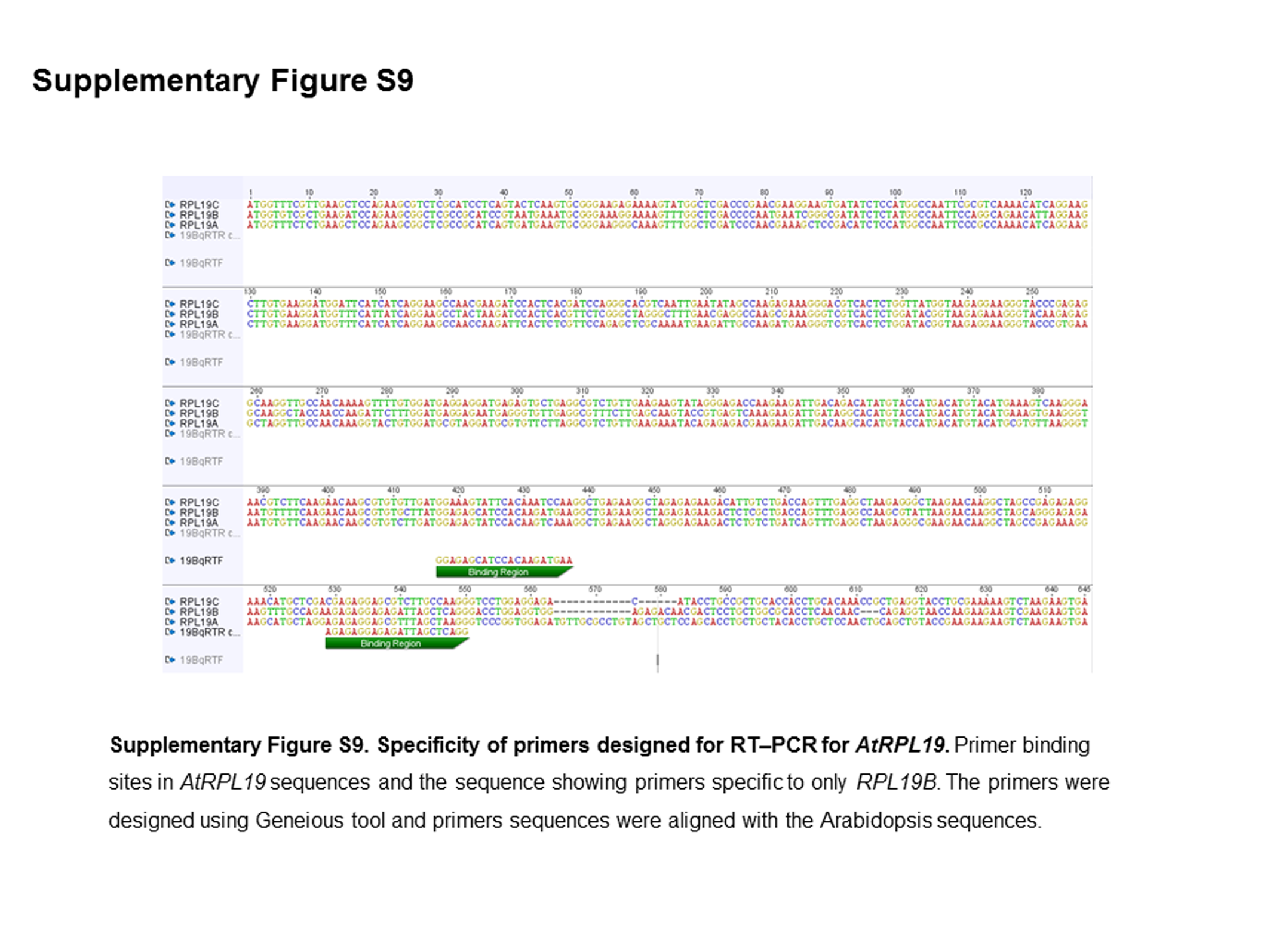

Supplement: Supplementary file 9 [file Image9.TIF]
