# Supplementary material for: Plant Ribosomal Proteins, RPL12 and RPL19, Play a Role in Nonhost Disease Resistance against Bacterial Pathogens
Source: Front Plant Sci. 2016 Jan 6;6:1192. doi: 10.3389/fpls.2015.01192 (PMC4702080; doi:10.3389/fpls.2015.01192)
Supplement: Supplementary file 10 [file Table1.DOCX]

**Supplementary Table S1.** Details of primers used in this study.

| Sl. No. | Name of the primer/gene | Primer sequence | Particulars |
| --- | --- | --- | --- |
| 1 | 19A05 RT F | CCAGGAGGGCAAAGAACAAA | For quantification of transcript levels under host/nonhost pathogen stress in *N. benthamiana* by real time RT-qPCR (not for VIGS down regulation) |
|  | 19A05 RT R | CCTGGTCCCTGTGCCAAAC |  |
| 2 | 14G03TH RT F | GCAGCGGCGCTCGTAA |  |
|  | 14G03TH RT R | CGTCTTTTTGCGATCTCTCTCA |  |
| 3 | NbRPL19SQRT F | AAAGGTGCTGTGGATGAGGAGGAT | Primers for to quantify VIGS downregulation of *NbRLP19* & *NbRLP12* by RT-qPCR. Designed to amplify outside region used in VIGS vector (semi quantitative RT-PCR for double silencing). |
|  | NbRPL19SQRT R | CTTCCTTTCCCTGCTGGCTTTGTT |  |
| 4 | NbRPL12SQRT R | CCAGAGGAATCTCAACATCACCATC |  |
|  | NbRPL12SQRT F | AAGGAGCCTGAGAGAGATCGCAAA |  |
