# Supplementary material for: Plant Ribosomal Proteins, RPL12 and RPL19, Play a Role in Nonhost Disease Resistance against Bacterial Pathogens
Source: Front Plant Sci. 2016 Jan 6;6:1192. doi: 10.3389/fpls.2015.01192 (PMC4702080; doi:10.3389/fpls.2015.01192)
Supplement: Supplementary file 11 [file Table2.DOCX]

**Supplementary Table S2. Statistical tables for two-way ANOVA and Fishers LSD test**

**Two-way ANOVA for bacterial growth at different time points for pathogen treated *N. benthamiana* plants shown in Figure 2**

***P. syringae* pv tomato T1**

| *Source of Variation* | *SS* | *d.f.* | *MS* | *F* | *p-level* | *F crit* | *Omega Sqr.* |
| --- | --- | --- | --- | --- | --- | --- | --- |
| *Factor #1 (1)* | 47.046 | 3.000 | 15.682 | 314.579 | 0.000 | 3.028 | 0.550 |
| *Factor #2 (1)* | 23.065 | 2.000 | 11.533 | 231.345 | 0.000 | 3.422 | 0.269 |
| *Factor #1 + #2 (1 x 1)* | 13.989 | 6.000 | 2.332 | 46.771 | 0.000 | 2.528 | 0.161 |
| *Within Groups* | 1.147 | 23.000 | 0.050 |  |  |  |  |
| *Total* | 85.247 | 34.000 | 2.507 |  |  |  |  |

Factor 1- Time points; Factor -2 – VIGS silenced RPL12/19 plants

***P. syringae* pv glycinea**

| *Source of Variation* | *SS* | *d.f.* | *MS* | *F* | *p-level* | *F crit* | *Omega Sqr.* |
| --- | --- | --- | --- | --- | --- | --- | --- |
| *Factor #1 (1)* | 63.18 | 3.00 | 21.06 | 115.98 | 0.00 | 3.03 | 0.75 |
| *Factor #2 (1)* | 7.34 | 2.00 | 3.67 | 20.22 | 0.00 | 3.42 | 0.08 |
| *Factor #1 + #2 (1 x 1)* | 8.70 | 6.00 | 1.45 | 7.99 | 0.00 | 2.53 | 0.09 |
| *Within Groups* | 4.18 | 23.00 | 0.18 |  |  |  |  |
| *Total* | 83.40 | 34.00 | 2.45 |  |  |  |  |
| *Omega squared for combined effect* | 0.92 |  |  |  |  |  |  |

***X. campestris* pv. vesicatoria**

| *Source of Variation* | *SS* | *d.f.* | *MS* | *F* | *p-level* | *F crit* | *Omega Sqr.* |
| --- | --- | --- | --- | --- | --- | --- | --- |
| *Factor #1 (1)* | 25.15 | 3.00 | 8.38 | 29.95 | 0.00 | 3.03 | 0.73 |
| *Factor #2 (1)* | 0.10 | 2.00 | 0.05 | 0.18 | 0.83 | 3.42 | 0.00 |
| *Factor #1 + #2 (1 x 1)* | 1.26 | 6.00 | 0.21 | 0.75 | 0.62 | 2.53 | 0.00 |
| *Within Groups* | 6.44 | 23.00 | 0.28 |  |  |  |  |
| *Total* | 32.96 | 34.00 | 0.97 |  |  |  |  |
| *Omega squared for combined effect* | 0.71 |  |  |  |  |  |  |

***P. syringae* pv tabaci**

| *Source of Variation* | *SS* | *d.f.* | *MS* | *F* | *p-level* | *F crit* | *Omega Sqr.* |
| --- | --- | --- | --- | --- | --- | --- | --- |
| *Factor #1 (1)* | 333.46 | 4.00 | 83.36 | 1150.32 | 0.00 | 2.70 | 0.96 |
| *Factor #2 (1)* | 1.73 | 2.00 | 0.86 | 11.90 | 0.00 | 3.33 | 0.00 |
| *Factor #1 + #2 (1 x 1)* | 8.54 | 8.00 | 1.07 | 14.73 | 0.00 | 2.28 | 0.02 |
| *Within Groups* | 2.10 | 29.00 | 0.07 |  |  |  |  |
| *Total* | 345.83 | 43.00 | 8.04 |  |  |  |  |
| *Omega squared for combined effect* | 0.99 |  |  |  |  |  |  |
|  |  |  |  |  |  |  |  |

**Two-way ANOVA for bacterial growth at different time points for dip inoculated *N. benthamiana* plants shown in Figure 3**

| *Source of Variation* | *SS* | *d.f.* | *MS* | *F* | *p-level* | *F crit* | *Omega Sqr.* |
| --- | --- | --- | --- | --- | --- | --- | --- |
| *Factor #1 (1)* | 21.92 | 2.00 | 10.96 | 82.70 | 0.00 | 3.59 | 0.48 |
| *Factor #2 (1)* | 12.35 | 2.00 | 6.18 | 46.60 | 0.00 | 3.59 | 0.27 |
| *Factor #1 + #2 (1 x 1)* | 8.71 | 4.00 | 2.18 | 16.43 | 0.00 | 2.96 | 0.18 |
| *Within Groups* | 2.25 | 17.00 | 0.13 |  |  |  |  |
| *Total* | 45.24 | 25.00 | 1.81 |  |  |  |  |
| *Omega squared for combined effect* | 0.92 |  |  |  |  |  |  |

Factor 1- Time points; Factor -2 – VIGS silenced RPL12/19 plants

**Fisher LSD test and Bonferroni test for differences between means for the disease severity caused by nonhost pathogen shown in Figure 4D**

**Bonferroni test for differences between means**

| *Alpha/N* | 0.008 |  |  |  |  |  |
| --- | --- | --- | --- | --- | --- | --- |
| *Group vs Group (Contrast)* | *Difference* | *95% Confidence Interval* | | *Test Statistics* | *p-level* | |
| 1 vs 2 | 46.456 | 22.962 | 69.950 | 5.573 | 0.000 | |
| 1 vs 3 | 37.770 | 14.276 | 61.264 | 4.531 | 0.000 | |
| 1 vs 4 | 26.924 | 3.430 | 50.417 | 3.230 | 0.018 | |
| 2 vs 3 | -8.686 | -31.479 | 14.106 | 1.074 | 1.000 | |
| 2 vs 4 | -19.533 | -42.325 | 3.260 | 2.415 | 0.131 | |
| 3 vs 4 | -10.847 | -33.639 | 11.946 | 1.341 | 1.000 | |

**Fisher LSD**

| *Group vs Group (Contrast)* | *Difference* | *Test Statistics* | *p-level* | *Accepted?* |  |  |
| --- | --- | --- | --- | --- | --- | --- |
| 1 vs 2 | 46.456 | 5.573 | 0.000 | *accepted* | |  |
| 1 vs 3 | 37.770 | 4.531 | 0.000 | *accepted* | |  |
| 1 vs 4 | 26.924 | 3.230 | 0.003 | *accepted* | |  |
| 2 vs 3 | -8.686 | 1.074 | 0.291 | *rejected* |  |  |
| 2 vs 4 | -19.533 | 2.415 | 0.021 | *accepted* | |  |
| 3 vs 4 | -10.847 | 1.341 | 0.189 | *rejected* |  |  |

Group

1-No symptom; 2- less disease; 3- moderate disease; 4- severe disease

**Two-way ANOVA for response of *RPL* genes after pathogen treatment at different time points shown in Figure 5.**

| *Source of Variation* | *SS* | *d.f.* | *MS* | *F* | *p-level* | *F crit* | *Omega Sqr.* |
| --- | --- | --- | --- | --- | --- | --- | --- |
| *Factor #1 (1)* | 0.18 | 3.00 | 0.06 | 0.97 | 0.43 | 3.03 | 0.00 |
| *Factor #2 (1)* | 0.11 | 2.00 | 0.05 | 0.87 | 0.43 | 3.42 | 0.00 |
| *Factor #1 + #2 (1 x 1)* | 0.33 | 6.00 | 0.06 | 0.88 | 0.53 | 2.53 | 0.00 |
| *Within Groups* | 1.45 | 23.00 | 0.06 |  |  |  |  |
| *Total* | 2.08 | 34.00 | 0.06 |  |  |  |  |
| *Omega squared for combined effect* | 0.00 |  |  |  |  |  |  |

***RPL19* gene expression**

Factor 1- Time points; Factor -2 – pathogen treatments

***RPL12* gene expression**

|  | *Source of Variation* | *SS* | *d.f.* | *MS* | *F* | *p-level* | *F crit* | *Omega Sqr.* |
| --- | --- | --- | --- | --- | --- | --- | --- | --- |
|  | *Factor #1 (1)* | 0.331 | 3 | 0.110 | 7.790 | 0.001 | 3.028 | 0.139 |
|  | *Factor #2 (1)* | 0.606 | 2 | 0.303 | 21.396 | 0.000 | 3.422 | 0.278 |
|  | *Factor #1 + #2 (1 x 1)* | 0.803 | 6 | 0.134 | 9.452 | 0.000 | 2.528 | 0.345 |
|  | *Within Groups* | 0.326 | 23 | 0.014 |  |  |  |  |
|  | *Total* | 2.066 | 34 | 0.061 |  |  |  |  |
|  | *Omega squared for combined effect* | 0.762 |  |  |  |  |  |  |
|  |  |  |  |  |  |  |  |  |
|  | *Factor #1 (1)* | Time point |  |  |  |  |  |  |
|  | *Factor #2 (1)* | Pathogen treatment |  |  |  |  |  |  |
|  | *Factor #1 + #2 (1 x 1)* | Interaction |  |  |  |  |  |  |

**Two-way ANOVA for bacterial growth at different time points when both NbRPL12 and NbRPL19 were silenced in *N. benthamiana* plants as shown in** **Figure 6C**

| *Source of Variation* | *SS* | *d.f.* | *MS* | *F* | *p-level* | *F crit* | *Omega Sqr.* |
| --- | --- | --- | --- | --- | --- | --- | --- |
| *Factor #1 (1)* | 0.11 | 1.00 | 0.11 | 1.34 | 0.29 | 5.59 | 0.00 |
| *Factor #2 (1)* | 22.24 | 1.00 | 22.24 | 268.54 | 0.00 | 5.59 | 0.94 |
| *Factor #1 + #2 (1 x 1)* | 0.68 | 1.00 | 0.68 | 8.20 | 0.02 | 5.59 | 0.03 |
| *Within Groups* | 0.58 | 7.00 | 0.08 |  |  |  |  |
| *Total* | 23.61 | 10.00 | 2.36 |  |  |  |  |
| *Omega squared for combined effect* | 0.96 |  |  |  |  |  |  |

Factor 1- Time points; Factor -2 – pathogen treatments

**Two-way ANOVA for bacterial growth at different time points in Arabidopsis Salk mutant plants as shown in Figures 7B & D.**

***P. syringae* pv tabaci**

| *Source of Variation* | *SS* | *d.f.* | *MS* | *F* | *p-level* | *F crit* | *Omega Sqr.* |
| --- | --- | --- | --- | --- | --- | --- | --- |
| *Factor #1 (1)* | 35.049 | 3.000 | 11.683 | 224.871 | 0.000 | 3.028 | 0.770 |
| *Factor #2 (1)* | 2.063 | 2.000 | 1.031 | 19.852 | 0.000 | 3.422 | 0.043 |
| *Factor #1 + #2 (1 x 1)* | 6.984 | 6.000 | 1.164 | 22.405 | 0.000 | 2.528 | 0.147 |
| *Within Groups* | 1.195 | 23.000 | 0.052 |  |  |  |  |
| *Total* | 45.291 | 34.000 | 1.332 |  |  |  |  |
| *Omega squared for combined effect* | 0.960 |  |  |  |  |  |  |

Factor 1- Time points; Factor -2 –Salk Mutant lines treated with pathogen

***P. syringae* pv tomato (DC3000)**

| *Source of Variation* | *SS* | *d.f.* | *MS* | *F* | *p-level* | *F crit* | *Omega Sqr.* |
| --- | --- | --- | --- | --- | --- | --- | --- |
| *Factor #1 (1)* | 58.49 | 3.00 | 19.50 | 645.80 | 0.00 | 3.03 | 0.97 |
| *Factor #2 (1)* | 0.20 | 2.00 | 0.10 | 3.38 | 0.05 | 3.42 | 0.00 |
| *Factor #1 + #2 (1 x 1)* | 1.10 | 6.00 | 0.18 | 6.06 | 0.00 | 2.53 | 0.02 |
| *Within Groups* | 0.69 | 23.00 | 0.03 |  |  |  |  |
| *Total* | 60.48 | 34.00 | 1.78 |  |  |  |  |
| *Omega squared for combined effect* | 0.98 |  |  |  |  |  |  |

Factor 1- Time points; Factor -2 –Salk Mutant lines treated with pathogen

**Two-way ANOVA for gene expression in VIGS silenced *N. benthamiana* plants shown in Supplementary Figure S1**

***NbRPL19* gene expression**

| *Source of Variation* | *SS* | *d.f.* | *MS* | *F* | *p-level* | *F crit* | *Omega Sqr.* |
| --- | --- | --- | --- | --- | --- | --- | --- |
| *Factor #1 (1)* | 0 | 0 | #N/A | #N/A | 0 | #N/A | 0 |
| *Factor #2 (1)* | 1.244 | 2 | 0.622 | 22.194 | 0.0032 | 5.786 | 0.841 |
| *Factor #1 + #2 (1 x 1)* | 0 | 0 | #N/A | #N/A | 0 | #N/A | 0 |
| *Within Groups* | 0.140 | 5 | 0.0280 |  |  |  |  |
| *Total* | 1.384 | 7 | 0.197 |  |  |  |  |
| *Omega squared for combined effect* | 0.841 |  |  |  |  |  |  |

Factor 1- GFP silenced plants; Factor -2 – NbRPL19

***NbRPL12* gene expression**

| *Source of Variation* | *SS* | *d.f.* | *MS* | *F* | *p-level* | *F crit* | *Omega Sqr.* |
| --- | --- | --- | --- | --- | --- | --- | --- |
| *Factor #1 (1)* | 0 | 0 | #N/A | #N/A | 0 | #N/A | 0 |
| *Factor #2 (1)* | 0.6 | 2 | 0.3 | 15 | 0.0077 | 5.786 | 0.778 |
| *Factor #1 + #2 (1 x 1)* | 0 | 0 | #N/A | #N/A | 0 | #N/A | 0 |
| *Within Groups* | 0.1 | 5 | 0.02 |  |  |  |  |
| *Total* | 0.7 | 7 | 0.1 |  |  |  |  |
| *Omega squared for combined effect* | 0.78 |  |  |  |  |  |  |

Factor 1- GFP silenced plants; Factor -2 – NbRPL12

**Two-way ANOVA for silenced plants treated with the host pathogen as shown in Supplementary Figure S4.**

| *Source of Variation* | *SS* | *d.f.* | *MS* | *F* | *p-level* | *F crit* | *Omega Sqr.* |
| --- | --- | --- | --- | --- | --- | --- | --- |
| *Factor #1 (1)* | 0 | 0 | #N/A | #N/A | 0 | #N/A | 0 |
| *Factor #2 (1)* | 403.50 | 2 | 201.751 | 1.20 | 0.374 | 5.786 | 0.0485 |
| *Factor #1 + #2 (1 x 1)* | 0 | 0 | #N/A | #N/A | 0 | #N/A | 0 |
| *Within Groups* | 837.80 | 5 | 167.561 |  |  |  |  |
| *Total* | 1241.30 | 7 | 177.329 |  |  |  |  |
| *Omega squared for combined effect* | 0.04853 |  |  |  |  |  |  |

Factor 1- GFP silenced plants; Factor -2–NbRPL19/12 silenced plants treated with host pathogen

**Two-way ANOVA for gene expression in individual and double gene silenced *N. benthamiana* plants shown in Supplementary Figure S5**

**A. *NbRPL12* gene expression**

| *Source of Variation* | *SS* | *d.f.* | *MS* | *F* | *p-level* | *F crit* | *Omega Sqr.* |
| --- | --- | --- | --- | --- | --- | --- | --- |
| *Factor #1 (1)* | 0 | 0 | #N/A | #N/A | 0 | #N/A | 0 |
| *Factor #2 (1)* | 1.444 | 3.000 | 0.481 | 42.027 | 0.000 | 4.347 | 0.918 |
| *Factor #1 + #2 (1 x 1)* | 0 | 0 | #N/A | #N/A | 0 | #N/A | 0 |
| *Within Groups* | 0.080 | 7.000 | 0.011 |  |  |  |  |
| *Total* | 1.524 | 10.000 | 0.152 |  |  |  |  |
| *Omega squared for combined effect* | 0.918 |  |  |  |  |  |  |

Factor 1- GFP silenced plants; Factor -2 – NbRPL19

**B. *NbRPL19* Gene expression**

| *Source of Variation* | *SS* | *d.f.* | *MS* | *F* | *p-level* | *F crit* | *Omega Sqr.* |
| --- | --- | --- | --- | --- | --- | --- | --- |
| *Factor #1 (1)* | 0 | 0 | #N/A | #N/A | 0 | #N/A | 0 |
| *Factor #2 (1)* | 2.11 | 3 | 0.70 | 64.46 | 0.00 | 4.35 | 0.95 |
| *Factor #1 + #2 (1 x 1)* | 0 | 0 | #N/A | #N/A | 0 | #N/A | 0 |
| *Within Groups* | 0.076 | 7 | 0.011 |  |  |  |  |
| *Total* | 2.185 | 10 | 0.218 |  |  |  |  |
| *Omega squared for combined effect* | 0.945 |  |  |  |  |  |  |

Factor 1- GFP silenced plants; Factor -2 – NbRPL12

Index:

SS: Sum of square

d.f: Degrees of freedom

MS: Means square

F: F-value

p-level: *p* value

F crit: F-value critical

Omega Sqr.: Omega square
